# Initiation of Breastfeeding in Low- and Middle-Income Countries: A Time-to-Event Analysis

**DOI:** 10.9745/GHSP-D-20-00361

**Published:** 2021-06-30

**Authors:** Lindsay Mallick, Wenjuan Wang, Shiza Farid, Thomas Pullum

**Affiliations:** a University of Maryland School of Public Health, College Park, MD, USA.; b Avenir Health, Glastonbury, CT, USA.; c ICF, Rockville, MD, USA.; d The Demographic and Health Surveys Program, Rockville, MD, USA.

## Abstract

**Objective:**

Early breastfeeding has numerous benefits for both the mother and her baby. Previous research typically analyzes breastfeeding initiation in binary terms (within the first hour or day). Although delays are associated with cesarean delivery and skin-to-skin contact may facilitate early breastfeeding, a more nuanced understanding of these relationships is needed.

**Methods:**

With data from 31 countries that had a Demographic and Health Survey since 2015, we described breastfeeding initiation among babies most recently born in the last 2 years to women aged 15–49 years. In a subset of 21 countries, we conducted survival analysis with multivariable log-logistic accelerated failure time (AFT) regressions to examine factors associated with time to initiation of breastfeeding, specifically the mode of delivery and skin-to-skin contact, controlling for receipt of health care as well as socioeconomic and demographic characteristics of mothers and babies.

**Findings:**

Babies in most countries began breastfeeding within a few hours after birth. The mean time to initiation of breastfeeding ranged from 1.7 hours in Burundi to 32 hours in Pakistan and 40 hours in Chad. In most countries (24 of 31), the median time was 0.5 hours. Median time to initiation was greater for births by cesarean delivery compared with vaginal births at health facilities. After controlling for covariates, AFT models showed significant delays in breastfeeding among cesarean deliveries in most countries, with as much as a 9-fold delay in Senegal. Immediate skin-to-skin contact was significantly associated with a shorter time to initiation.

**Conclusion:**

Efforts to promote early breastfeeding should encourage skin-to-skin and target cesarean deliveries.

## Abstract (precis)

This article uses country-specific data to provide information for stakeholders about delays in breastfeeding, especially for babies born via cesarean delivery, and provide evidence to support skin-to-skin contact to promote early breastfeeding.

## Teaser

**Key Findings:**

- We calculated a continuous measure of time to initiation of breastfeeding in low- and middle-income countries using recent Demographic and Health Surveys Program data and found that although the average time ranged from 1.7 hours in Burundi to 40 hours in Chad, the median time to initiation met the benchmark of within 1 hour in most countries.
- In nearly all countries studied and after controlling for confounding factors, cesarean delivery was associated with a significant delay in median time to initiation of breastfeeding, ranging from 30% to 830% longer time compared with vaginal deliveries in facilities. Conversely, immediate skin-to-skin contact was associated with an earlier time to initiation in almost all countries (10%–80% earlier).

**Key Implications:**

- Program managers and hospital administrators should consider adopting facility-based programs that encourage early breastfeeding, such as the Baby-Friendly Hospital Initiative, particularly in places where breastfeeding is substantially delayed.
- Government officials should endorse health facility-based policies that promote breastfeeding practices such as skin-to-skin and provide health care worker training opportunities to build awareness of these practices.

## INTRODUCTION

Breastfeeding has prodigious benefits for both the mother and baby such that it protects infants against infections, supports the growth of the child, and protects mothers from postpartum hemorrhage and some types of cancer.^1^^,^^2^ Owing to its protective mechanisms, breastfeeding can avert nearly 1 million deaths of mothers and children each year.^1^

Early initiation of breastfeeding (EIBF), defined as the initiation of breastfeeding within the first hour after delivery, is particularly beneficial. The early initiation triggers the release of hormones that help the mother's uterus contract and thus prevent hemorrhage.^3^^,^^4^ Not only does EIBF provide early milk (colostrum), which has additional protective benefits for the baby, it also encourages future milk production.^5^ Research has also identified a reduced risk of neonatal mortality with EIBF.^6^^–^^8^

Immediate breastfeeding and skin-to-skin contact are intricately related; skin-to-skin may facilitate spontaneous breastfeeding by the newborn and plays an important role in breastfeeding outcomes.^9^^–^^13^ Early breastfeeding, in addition to skin-to-skin contact, provides thermal care for the newborn.^14^^,^^15^ The World Health Organization (WHO) recommends that both breastfeeding and skin-to-skin should begin within the first hour after birth.^16^ Breastfeeding in the first hour after birth is further considered “essential newborn care.”^17^

Initiation of breastfeeding can be delayed by individual factors, conditions of the birth (e.g., preterm, low birthweight), cultural influences, or barriers at the health facility, including complications during vaginal and cesarean deliveries.^1^^,^^18^^–^^20^ WHO recommends that breastfeeding begin as soon as possible after cesarean delivery given the importance of early breastfeeding.^16^ With properly trained, supportive health workers, women can be successful in this endeavor. The Baby-Friendly Hospital Initiative encourages provider training on breastfeeding.^16^

To inform these practices, a nuanced understanding of the delay in breastfeeding following cesarean delivery is warranted, yet most research to date defines and analyzes breastfeeding initiation in binary terms—within the first hour or the first day. A more nuanced description of delays in breastfeeding, especially if substantial differences are seen by mode of delivery, could provide insight for targeted policies or programs. The objectives of this article are first to examine the time to initiation of breastfeeding in a more granular way than existing research and, second, to compare the timing of initiation by factors that may influence early breastfeeding, specifically mode of delivery and skin-to-skin contact.

A more nuanced description of delays in breastfeeding, especially if substantial differences are observed by mode of delivery, could provide insight for targeted policies or programs.

## DATA

Our analysis used data from 200,054 births across 31 countries where the Demographic and Health Surveys (DHS) Program has conducted surveys since 2015 to describe breastfeeding initiation. We used a subset of these countries (21) with information about skin-to-skin, a potential determinant of early breastfeeding, to further examine the factors associated with time to initiation of breastfeeding among babies most recently born in the last 2 years to women aged 15–49 years. For the most recent birth, mothers were asked if they ever breastfed and, if so, about the timing of initiation of breastfeeding: “How long after birth did you first put (NAME) to the breast?” Women were prompted to respond in either hours or days after birth. All surveys conducted since January 2015 and released before September 2019 that included this question were included in the descriptive analysis. Table 1 presents the total number of births analyzed in each country, as well as the percentage of babies born via cesarean delivery.

**TABLE 1.** Sample of Most Recent Live-Born Children in the 2 Years Before Each Demographic Health Survey, 2014–2018

Region	Country	Cesarean Delivery, No. (%)	All Births, No.
North Africa, West and Central Asia, Europe	Albania 2017–2018	328 (31.7)	1,034
	Armenia 2015–2016	143 (21.5)	664
	Egypt 2014	3,602 (57.4)	6,271
	Jordan 2017–2018	955 (27.7)	3,452
	Maldives 2016–2017	460 (42.8)	1,074
	Tajikistan 2017	145 (5.9)	2,465
South and Southeast Asia	Bangladesh 2014	780 (24.6)	3,166
	Cambodia 2014	236 (8.1)	2,906
	India 2015–2016	17,838 (19.3)	92,600
	Indonesia 2017	1,260 (19.2)	6,561
	Myanmar 2015–2016	350 (21.2)	1,652
	Nepal 2016	198 (10.1)	1,965
	Pakistan 2017–2018	998 (25.8)	3,864
	Philippines 2017	572 (15.5)	3,693
	Timor-Leste 2016	97 (3.5)	2,815
Sub-Saharan Africa	Angola 2015–2016	203 (3.8)	5,298
	Benin 2017–2018	265 (4.9)	5,405
	Burundi 2016–2017	282 (5.2)	5,368
	Chad 2014–2015	101 (1.5)	6,656
	Ethiopia 2016	110 (2.6)	4,244
	Ghana 2014	276 (12.4)	2,234
	Kenya 2014	288 (8.2)	3,496
	Lesotho 2014	136 (10.1)	1,348
	Malawi 2015–2016	435 (6.6)	6,579
	Senegal 2016	251 (5.7)	4,410
	South Africa 2016	337 (24.7)	1,364
	Tanzania 2015–2016	268 (6.5)	4,106
	Uganda 2016	414 (7.1)	5,797
	Zimbabwe 2015	147 (6.1)	2,421
Latin America and Caribbean	Guatemala 2014–2015	1,403 (29.5)	4,756
	Haiti 2016–2017	136 (5.7)	2,390

## METHODS

We used several approaches to explore time to initiation of breastfeeding. First, we examined time to initiation of breastfeeding categorically for all babies. Based on a common distribution of time to initiation across countries, we created 7 categories of timing: within the first hour; 1–2 hours; 3–5 hours; 6–23 hours; the day after birth; 2–4 days; and 5 days or more, never breastfed, and don't know or missing.

We created a continuous variable of time to initiation using a commonly applied demographic method of converting discrete time data to continuous data.^21^^,^^22^ This continuous variable was used to calculate the mean time to initiation in each country for all births, and by mode and place of delivery. In DHS surveys, interviewers record the time to initiation in intervals of completed hours or days, where a response of “immediately” is recoded as 0; a response of 1 hour assumes no less than 1 hour and is thus outside of the benchmark for “within 1 hour.”^23^ If a woman reported beginning breastfeeding 1 hour after birth, this means she began breastfeeding no sooner than 60 minutes after birth and up to 119 minutes after birth. Although it is unlikely that retrospective self-report of time to initiation is so precise,^24^ an average for all women beginning within this interval (at least 1 completed hour after birth) would be likely to fall near 90 minutes.

In our analysis, we adjusted for this approximation by assigning the midpoint of the interval reported. For example, if a woman reported she began breastfeeding either immediately or within the first hour, we assigned the value of 0.5 hours, which represents the midpoint of the first hour. If she reported 1 hour, her response is converted to 1.5 hours. We converted responses in days to hours by multiplying by 24 and assuming the midpoint of the day; for example, a response of 1 day was coded as the midpoint between 1 day and 2 days in hours (36 hours). We calculated the mean and median time to breastfeeding among all births and by mode of delivery among ever-breastfed babies with nonmissing responses. We estimated the lower and upper bounds of the 95% confidence interval (CI) of the mean according to a Poisson distribution.

We conducted multivariable survival analyses to identify the factors associated with time to initiation of breastfeeding. Because skin-to-skin contact is an important factor in breastfeeding, we analyzed data from 21 of the 31 recent DHS surveys completed that included a question about skin-to-skin contact. The model specification was determined after assessing the proportional hazard assumption based on Schoenfeld residuals, which revealed that survival (time to initiation of breastfeeding) between covariate groups did not maintain proportionality over time and that relationships between covariates and the outcome were not consistently linear.^25^ Therefore, we selected an accelerated failure time (AFT) model, which does not require an assumption of proportional hazards.^26^^,^^27^ We tested 3 distributions of the AFT model (Weibull, log-normal, and log-logistic) for goodness of fit using Akaike's information criterion (AIC). We selected the log-logistic model because it produced the best fit model in every country but 2, wherein the AIC did not vary substantially between log-normal and log-logistic distributions.

Each model was restricted to babies who ever breastfed, starting as early as immediately after delivery, and did so within 4 days after birth. Data were censored at 4 days because, at that point, most women have reached lactogenesis stage II in which the composition of breastmilk has evolved from colostrum to transitional milk^28^ and when nearly all (99% or more in most countries) babies who ever breastfed had initiated breastfeeding. All analyses excluded babies who died within the first 4 days (between 0 and 35 babies per country) because these newborns may have had complications that inhibited their ability to breastfeed.^13^^,^^18^ We conducted 2 sensitivity analyses in which we removed these restrictions from the models to examine whether these exclusions (neonates who died within 4 days or babies who began breastfeeding after 4 days) altered our findings.

The models included socioeconomic, demographic, and health behavior characteristics of the mother and baby. Socioeconomic and demographic characteristics of the mother included place of residence (urban and rural); region; wealth quintile; education (none, primary, secondary or higher); employment (not employed, employed-professional, and employed-manual, agricultural, or other); exposure to mass media (less than once per week and once per week and more); and parity (1, 2, 3, 4+ live births). We also included marital status (currently married, not currently married). Jordan and Pakistan sampled only ever-married women. In the Republic of Maldives DHS, there was no designation for urban and rural within atolls and regions, so it was only possible to include region (and not place of residence) due to collinearity. Child characteristics included sex and birth size. Birth size was categorized as small, average, or above average, based on the weight of the child if available or recalled, or the mother's perception in the absence of a reported weight. Health behavior and care-related variables included antenatal care visits (<4, 4+); mode and place of delivery (vaginal delivery at home, vaginal delivery in facility, cesarean delivery in facility); whether the baby was placed on the chest immediately after birth (had immediate skin-to-skin contact, either no or yes); and whether a postnatal check was done within 1 hour for either the mother or baby (no or yes). The postnatal check was based on the mother's report of whether anyone checked her or the baby's health within the first hour after delivery.

We used Stata version 16.0 for the analysis. All statistical tests adjusted for the complex survey design using multistage probability samples drawn from an existing sample frame and applied survey weights to account for nonresponse and disproportionate sampling.^23^

## RESULTS

**FIGURE 1:**
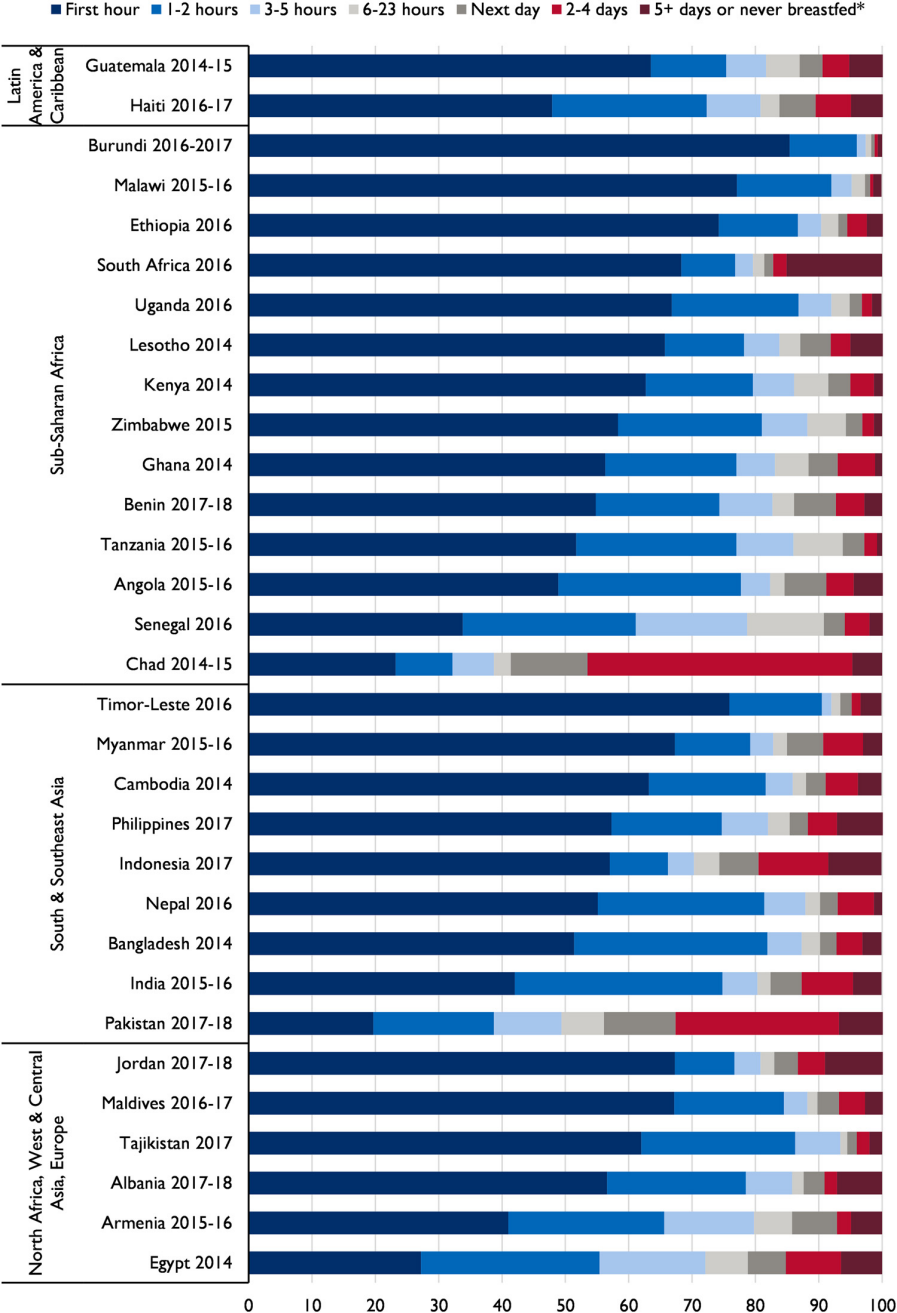
Percentage Distribution of Children by Time to Initiation of Breastfeeding Among Most Recent Live-Born Children in the 2 Years Before the Survey, 2014–2018 *Category includes “don't know” or missing responses.

Figure 1 presents the distribution of time to initiation of breastfeeding in 7 categories with additional information (percentage and 95% CI for each category in each country) provided in Supplement Table 1. In nearly all countries, 80% of babies began breastfeeding within the first day after birth, except Chad and Pakistan, where only 41% and 56% of babies began breastfeeding on the first day, respectively. Fewer than 10% of babies never breastfed or began breastfeeding 5 days after birth or later, the only exception to this being South Africa (15%). Although the majority of babies in most countries began breastfeeding immediately (within the first hour after birth), substantial differences existed across and within regions. For example, in sub-Saharan Africa, 85% of babies in Burundi began breastfeeding immediately versus 23% in Chad. We found inconsistent practice in South and Southeast Asia, where EIBF ranged from 20% in Pakistan to 76% in Timor-Leste, and in North Africa, West and Central Asia, and Europe, from 27% in Egypt to 67% in Jordan.

Although the majority of babies in most countries began breastfeeding within the first hour after birth, substantial differences existed across and within regions.

Table 2 shows the mean and median time to initiation of breastfeeding in hours for all babies and by mode of delivery, highlighting the disparities in time to initiation by mode of delivery. For all babies, the mean time ranged from 1.7 hours in Burundi to 40 hours in Chad. The mean time was less than 7 hours in more than half of the countries among babies born by vaginal delivery at a health facility but typically greater than 20 hours for those born by cesarean delivery. The median time was half an hour after delivery among all births as well as for vaginal deliveries at home and at a health facility. Among cesarean deliveries, the median time to initiation was 2.5 hours or more in most countries. On average, a cesarean delivery appeared to delay breastfeeding the least in the Republic of Maldives and South Africa and the most in Senegal. In the region where cesarean delivery was most common (West and Central Asia and Europe), cesarean delivery was the least delayed compared with vaginal births.

**TABLE 2.** Mean and Median Times (Hours) to Initiation of Breastfeeding, Among All Deliveries, Vaginal at Home, Vaginal at Facility, and Cesarean Delivery, Among Ever-Breastfed Last-Born Children Born in the Past 2 Years

Region	Country	All		Vaginal Delivery-Home		Vaginal Delivery-Facility		Cesarean Delivery	
		Mean (95% CI)	Med.	Mean (95% CI)	Med.	Mean (95% CI)	Med.	Mean (95% CI)	Med.
North Africa, West and Central Asia, Europe	Albania 2017–2018	5.3 (3.8, 7.5)	0.5	1.2 (0.0, 165.3)	0.5	3.5 (1.7, 7.0)	0.5	9.5 (7.0, 12.8)	1.5
	Armenia 2015–2016	8.0 (6.5, 9.9)	1.5	1.9 (0.0, 148.1)	2.5	5.0 (3.9, 6.4)	1.5	19.9 (13.9, 28.6)	5.5
	Egypt 2014	16.5 (15.2, 17.8)	2.5	12.2 (9.8, 15.2)	1.5	10.1 (8.7, 11.7)	1.5	20.9 (19.1, 22.8)	3.5
	Jordan 2017–2018	8.6 (7.2, 10.2)	0.5	2.5 (0.9, 7.0)	0.5	4.7 (3.6, 6.2)	0.5	19.6 (16.0, 24.1)	0.5
	Maldives 2016–2017	10.0 (6.6, 15.1)	0.5	12.8 (5.4, 30.6)	1.5	9.5 (4.4, 20.6)	0.5	10.2 (7.1, 14.6)	0.5
	Tajikistan 2017	3.7 (3.0, 4.6)	0.5	1.5 (1.0, 2.2)	0.5	2.6 (2.1, 3.2)	0.5	23.5 (15.2, 36.2)	3.5
South and Southeast Asia	Bangladesh 2014	7.4 (6.1, 8.8)	0.5	4.7 (3.7, 5.9)	0.5	5.1 (3.5, 7.3)	0.5	15.4 (11.6, 20.5)	1.5
	Cambodia 2014	7.6 (6.6, 8.9)	0.5	11.6 (8.3, 16.3)	0.5	4.9 (4.0, 5.8)	0.5	31.6 (24.3, 41.1)	3.5
	India 2015–2016	12.7 (12.3, 13.1)	1.5	15.9 (15.2, 16.7)	1.5	8.8 (8.4, 9.3)	1.5	22.4 (21.1, 23.7)	1.5
	Indonesia 2017	17.8 (16.6, 19.2)	0.5	18.6 (16.0, 21.6)	0.5	13.3 (12.0, 14.7)	0.5	33.0 (29.5, 36.9)	3.5
	Myanmar 2015–2016	11.7 (9.8, 13.9)	0.5	10.9 (8.6, 13.7)	0.5	11.0 (7.4, 16.3)	0.5	14.6 (10.6, 20.1)	0.5
	Nepal 2016	7.9 (6.5, 9.6)	0.5	9.6 (7.3, 12.6)	1.5	3.9 (2.9, 5.3)	0.5	24.2 (16.6, 35.3)	2.5
	Pakistan 2017–2018	31.7 (28.9, 34.7)	5.5	26.8 (23.3, 30.8)	3.5	24.2 (21.2, 27.6)	3.5	50.8 (43.6, 59.1)	36.0
	Philippines 2017	8.2 (6.8, 9.8)	0.5	6.1 (4.2, 8.9)	0.5	5.6 (4.5, 7.0)	0.5	21.9 (15.9, 30.2)	1.5
	Timor-Leste 2016	2.8 (2.3, 3.4)	0.5	2.4 (1.8, 3.1)	0.5	2.4 (1.8, 3.2)	0.5	14.4 (10.0, 20.9)	0.5
Sub-Saharan Africa	Angola 2015–2016	8.5 (7.4, 9.7)	0.5	8.0 (6.8, 9.3)	1.5	7.4 (5.6, 9.7)	0.5	26.8 (21.0, 34.4)	6.5
	Benin 2017–2018	9.0 (8.1, 9.9)	0.5	8.1 (6.5, 10.0)	0.5	8.0 (7.1, 9.0)	0.5	28.0 (22.5, 34.9)	5.5
	Burundi 2016–2017	1.7 (1.4, 2.3)	0.5	1.4 (0.8, 2.2)	0.5	1.3 (0.9, 1.7)	0.5	10.4 (6.6, 16.6)	1.5
	Chad 2014–2015	40.4 (38.0, 42.9)	36.0	39.9 (37.1, 42.9)	36.0	40.4 (37.5, 43.6)	36.0	66.4 (52.3, 84.3)	60.0
	Ethiopia 2016	5.4 (4.3, 6.7)	0.5	4.6 (3.4, 6.2)	0.5	5.0 (3.6, 7.0)	0.5	28.8 (14.3, 58.1)	1.5
	Ghana 2014	11.5 (8.2, 16.1)	0.5	9.2 (6.9, 12.4)	0.5	8.7 (5.2, 14.5)	0.5	30.9 (15.8, 60.3)	2.5
	Kenya 2014	8.2 (6.0, 11.2)	0.5	12.3 (7.0, 21.8)	0.5	4.5 (3.6, 5.6)	0.5	16.9 (11.3, 25.4)	2.5
	Lesotho 2014	8.7 (6.7, 11.2)	0.5	10.3 (6.8, 15.7)	0.5	6.4 (4.7, 8.7)	0.5	21.7 (10.8, 43.6)	3.5
	Malawi 2015–2016	2.1 (1.8, 2.5)	0.5	2.5 (1.7, 3.6)	0.5	1.7 (1.4, 2.0)	0.5	7.5 (4.4, 12.6)	0.5
	Senegal 2016	9.0 (8.0, 10.1)	2.5	6.7 (5.7, 7.9)	2.5	6.2 (5.4, 7.2)	1.5	53.4 (44.3, 64.2)	36.0
	South Africa 2016	4.5 (3.3, 6.2)	0.5	15.2 (3.9, 59.6)	0.5	3.4 (2.4, 4.8)	0.5	6.2 (3.6, 10.7)	0.5
	Tanzania 2015–2016	5.1 (4.5, 5.8)	0.5	6.8 (5.8, 8.0)	1.5	3.3 (2.6, 4.1)	0.5	11.9 (8.3, 16.8)	4.5
	Uganda 2016	4.2 (3.6, 5.0)	0.5	4.5 (3.3, 6.1)	0.5	3.0 (2.4, 3.8)	0.5	15.2 (10.0, 23.2)	1.5
	Zimbabwe 2015	4.6 (3.8, 5.6)	0.5	7.7 (5.7, 10.4)	1.5	3.0 (2.1, 4.1)	0.5	16.0 (12.4, 20.6)	3.5
Latin America and Caribbean	Guatemala 2014–2015	18.5 (15.4, 22.1)	0.5	7.1 (5.3, 9.5)	0.5	11.1 (8.4, 14.6)	0.5	40.9 (32.0, 52.2)	3.5
	Haiti 2016–2017	11.7 (10.0, 13.7)	1.5	8.9 (7.3, 10.8)	1.5	10.4 (8.0, 13.6)	0.5	52.7 (36.1, 77.0)	6.5

Abbreviations: CI, confidence interval; Med., median.

Supplement Tables 2a and 2b present the background characteristics and care-seeking behavior of mothers and babies included in the survival analysis. Skin-to-skin contact was a common practice in most countries in Europe and Asia (except for Pakistan, where only 8% of births had immediate skin-to-skin contact) but varied substantially across sub-Saharan Africa and Haiti.

**FIGURE 2:**
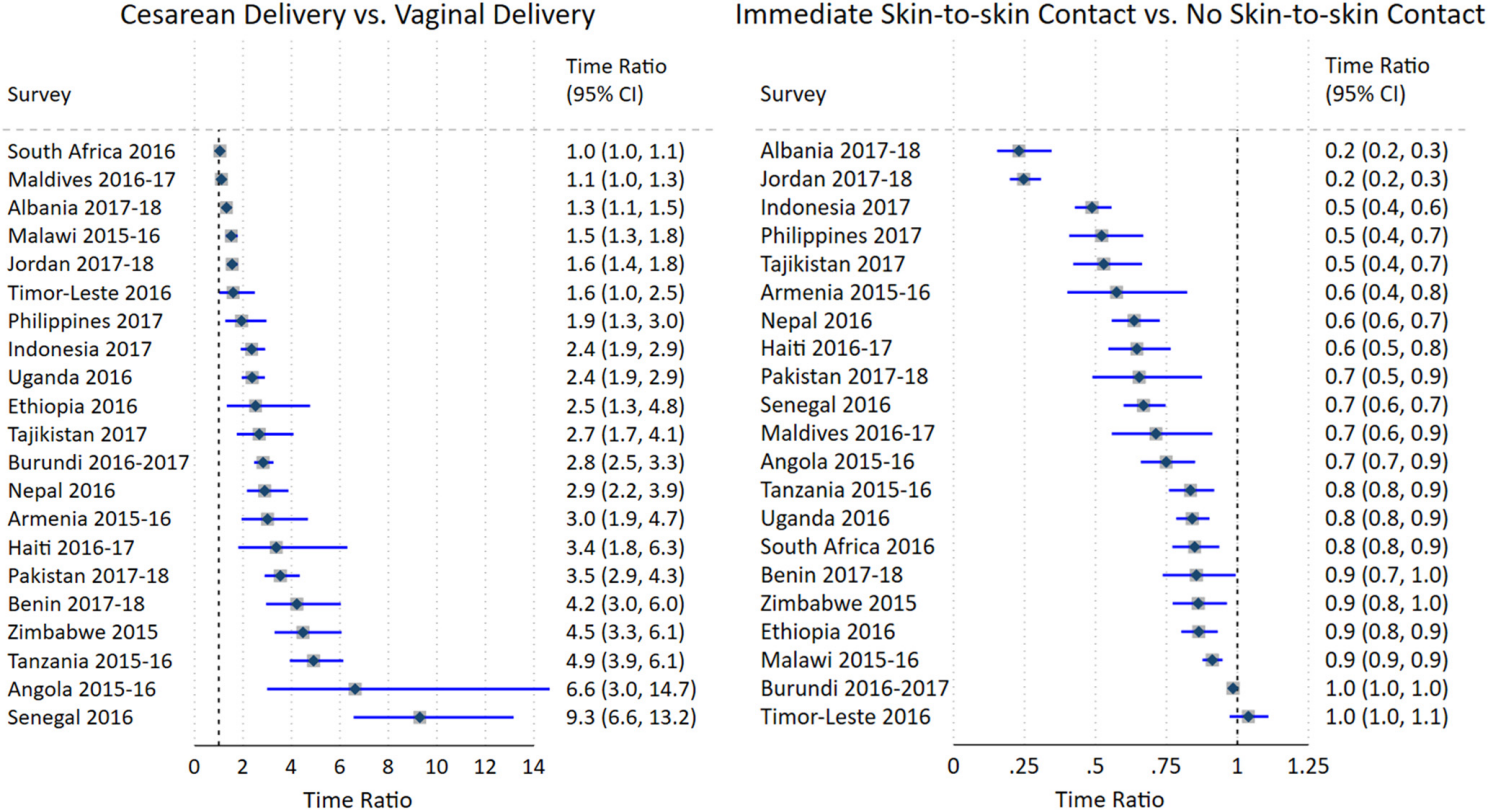
Time Ratios of Time to Initiation of Breastfeeding for Cesarean Delivery and Immediate Skin-to-skin Contact^a^ ^a^The reference group for cesarean delivery was vaginal deliveries in a facility and the reference for immediate skin-to-skin contact was no immediate skin-to-skin.

Figure 2 shows the time ratios (TRs) of 2 variables included in survival analysis: (1) cesarean delivery compared with vaginal delivery at facilities and (2) immediate skin-to-skin contact compared with no immediate skin-to-skin contact, after controlling for covariates of interest. Complementary Kaplan-Meier survival curves depicting these relationships are presented in Supplement Figures 1a, 1b, 2a, and 2b. With all other covariates held constant, compared with babies born vaginally in a facility, the median time to initiation of breastfeeding was significantly later among babies born via cesarean in all countries except the Maldives and South Africa. Stated differently, babies who were born vaginally had an earlier initiation of breastfeeding compared with babies that were born via cesarean delivery in almost all countries. Notably, the median time to initiation of breastfeeding among babies born through cesarean delivery was over 9 times slower than babies delivered vaginally at facilities in Senegal (TR: 9.3; 95% CI=6.6, 13.2), 6.6 times as long in Angola (95% CI=3.0, 14.7), and almost 5 times later in Tanzania (TR: 4.9; 95% CI=3.9, 6.1). As seen in Supplement Tables 3a and 3b, which include the TRs and 95% CIs for the full model for each country, in 7 countries (mostly in sub-Saharan Africa), there was a significant but less substantial delay in breastfeeding among babies born vaginally at home versus in a health facility.

Babies who were born vaginally had an earlier initiation of breastfeeding than babies born via cesarean delivery in almost all countries.

Conversely, skin-to-skin contact was significantly associated with a shorter time to initiation in all countries except Burundi and Timor-Leste. In most countries, the median time to initiation was 20% to 90% sooner among babies who received immediate skin-to-skin contact compared with babies whose mothers did not report immediate skin-to-skin contact. In Jordan and Albania, the time ratios were the most extreme (TRs 0.2; 95% CI=0.2, 0.3). The sensitivity analyses found no meaningful changes in the magnitude or strength of the associations after removing restrictions for neonatal survival and breastfeeding within 4 days.

## DISCUSSION

The benefits of EIBF have been well documented. One systematic review has also shown a dose-response relationship between the time to breastfeeding initiation and neonatal mortality: later initiation was associated with a greater risk of neonatal death.^8^ Given such evidence, using nationally representative samples from low- and middle-income countries, we assessed time to breastfeeding initiation in 31 countries and its determinants in 21 countries.

Levels of EIBF generally appeared higher than estimates by WHO and UNICEF based on data from household surveys conducted in 2016 or earlier.^29^ This finding suggested an increasing trend. However, in one-quarter of all countries studied, nearly half or more of all newborns were not breastfed until after the first hour, which is a delay that reduces the life-saving benefits of breastfeeding.^1^ Even when optimal early initiation cannot be achieved, breastfeeding within 24 hours of birth still protects newborns from a greater risk of neonatal mortality compared with initiation after 24 hours.^8^ Yet in 4 countries in this analysis, over 20% of babies began breastfeeding after 24 hours of delivery. For example, the median time to initiation in Pakistan was 36 hours. Research has identified a wide range of factors associated with late initiation of breastfeeding in Pakistan including the mother's working status and education, perceived benefit of breastfeeding, and traditional feeding practices.^30^

As in other studies,^18^^,^^20^ our findings demonstrated that cesarean delivery significantly delayed breastfeeding in almost all countries. This finding is concerning because the use of cesarean delivery has increased globally.^31^ Although cesarean delivery remains less common in most African countries, it has become more widely experienced by wealthier or more educated women.^24^ Despite the challenges faced by women after surgery, studies have shown that with proper support, initiation of breastfeeding within the first hour is possible for babies born by cesarean delivery.^2^^,^^29^^,^^32^ Also consistent with the findings in other studies, including those that used older DHS surveys and studies with an experimental or quasi-experimental design,^10^^,^^12^^,^^13^ skin-to-skin contact between the mother and her baby was associated with a shorter time to breastfeeding initiation in almost all countries. Immediate skin-to-skin contact is believed to be particularly important for newborns born by cesarean delivery for EIBF as well as exclusive breastfeeding.^32^^,^^33^

Our finding that cesarean delivery significantly delayed breastfeeding is concerning because this mode of delivery has increased globally.

Although EIBF and other breastfeeding practices could still be hampered by social and cultural beliefs or norms, the sizable increase in the coverage of facility delivery in low- and middle-income countries provides opportunities to promote optimal breastfeeding practices through interventions in health facilities. It is important to have current national guidelines that emphasize the importance of EIBF and essential training for health care staff. Training for health care staff has been associated with improved staff knowledge, attitude, and compliance with the recommended breastfeeding practices and with increased exclusive breastfeeding in some settings.^34^ Training for antenatal care providers on breastfeeding counseling has also been shown to relate to EIBF.^35^ Further research is needed to identify effective interventions that motivate health providers to promote EIBF.

### Limitations

This analysis has several limitations. First, self-reporting of the outcome variable is subject to recall bias. Previous research suggests that self-reports of the timing of initiation of breastfeeding (specifically, within 1 hour) do not meet acceptable validity criteria.^24^ Although women's reporting of EIBF overestimate observed EIBF, self-report still accurately reflects that early breastfeeding is more common among vaginal deliveries than cesarean deliveries.^36^ Our study attempted to minimize recall bias to the extent possible by restricting the analysis to the most recent birth in the past 2 years. Our study also assumes the midpoint of the interval reported, which may result in additional bias; however, for the majority of women who reported breastfeeding with 1 hour, shifting coding from 0 hours to 0.5 hours may more accurately reflect the timing of early initiation as babies progress through several initial phases of first relaxing, awakening, and activity before suckling.^9^

Further, our analysis could not account for all the complications that could interfere with breastfeeding. We controlled for birthweight as a proxy for preterm birth, although other complications could hinder early breastfeeding. For newborns, potential complications include congenital deformities, low Apgar scores, and near-miss cases, in which a pregnant woman comes close to maternal death. For mothers, complications can include eclampsia, anesthesia, blood transfusion, other intensive or surgical care such as hysterectomy, or other underlying conditions.^7^^,^^18^

## CONCLUSION

Although breastfeeding within the first several hours after birth is common in the 31 countries analyzed in this study, this analysis demonstrated consistent and often substantial lags among babies born by cesarean delivery but earlier time to breastfeeding initiation among babies with immediate skin-to-skin contact. Interventions that reduce the time to initiation of breastfeeding, such as skin-to-skin contact, should be targeted to health care systems given the increase in health facility delivery and cesarean delivery. Programs and policies should address country-specific practices, including the practice of and the delay in breastfeeding related to cesarean delivery.

## Supplementary Material

20-00361-Mallick-Supplement.pdf

## Notes

**Peer Reviewed**

**First published online:** May 17, 2021.
