# Supplementary material for: Initiation of Breastfeeding in Low- and Middle-Income Countries: A Time-to-Event Analysis
Source: Glob Health Sci Pract. 2021 Jun 30;9(2):308–17. doi: 10.9745/GHSP-D-20-00361 (PMC8324198; doi:10.9745/GHSP-D-20-00361)

Supplement to: Mallick L, Wang W, Farid S, Pullum T. Initiation of Breastfeeding in Low- and Middle-Income Countries: A Time-to-Event Analysis. *Glob Health Sci Pract*. 2021;9(2). https://doi.org/10.9745/GHSP-D-20-00361

## SUPPLEMENT TABLES AND FIGURES

| | |
|---|---|
| Supplement Table 1 | Time to initiation of breastfeeding, estimate and 95% confidence interval |
| Supplement Table 2a | Background characteristics of women included in the survival analysis, by country, Europe and Asia |
| Supplement Table 2b | Background characteristics of women included in the survival analysis, by country, sub-Saharan Africa and Haiti |
| Supplement Table 3a | Time ratio (TR) and 95% confidence intervals (CI) according to multivariable log-logistic regressions of time to initiation of breastfeeding, Europe and Asia |
| Supplement Table 3b | Time ratio (TR) and 95% confidence intervals (CI) according to multivariable log-logistic regressions of time to initiation of breastfeeding, sub-Saharan Africa and Haiti |
| Supplement Figure 1a. | Time to initiation of breastfeeding by place and mode of delivery, Europe and Asia |
| Supplement Figure 1b. | Time to initiation of breastfeeding by place and mode of delivery, sub-Saharan Africa and Haiti |
| Supplement Figure 2a. | Time to initiation of breastfeeding by immediate skin-to-skin contact, Europe and Asia |
| Supplement Figure 2b. | Time to initiation of breastfeeding by immediate skin-to-skin contact, sub-Saharan Africa and Haiti |

Supplement to: Mallick L, Wang W, Farid S, Pullum T. Initiation of Breastfeeding in Low- and Middle-Income Countries: A Time-to-Event Analysis. *Glob Health Sci Pract*. 2021;9(2).
**https://doi.org/10.9745/GHSP-D-20-00361**

**Supplement Table 1. Time to initiation of breastfeeding, estimate and 95% confidence interval**

| Region | Country | First hour | 1-2 hours | 3-5 hours | 6-23 hours | Next day | 2-4 days | 5+ days, never breastfed, missing[1] |
|---|---|---|---|---|---|---|---|---|
| North Africa, West & Central Asia, Europe | Albania 2017-18 | 56.6 (51.9,61.2) | 21.9 (18.2,26.2) | 7.3 (5.6,9.4) | 1.8 (1.1,3.0) | 3.3 (1.9,5.7) | 2.0 (1.2,3.2) | 7.1 (5.3,9.4) |
| | Armenia 2015-16 | 41.0 (36.7,45.5) | 24.6 (21.2,28.4) | 14.2 (11.3,17.7) | 6.0 (4.1,8.6) | 7.1 (5.2,9.5) | 2.2 (1.3,3.6) | 4.9 (3.4,7.1) |
| | Egypt 2014 | 27.2 (25.7,28.8) | 28.2 (26.9,29.6) | 16.7 (15.6,17.9) | 6.7 (6.0,7.5) | 6.0 (5.2,6.8) | 8.7 (7.8,9.6) | 6.5 (5.8,7.3) |
| | Jordan 2017-18 | 67.3 (64.7,69.8) | 9.4 (7.9,11.2) | 4.1 (3.3,5.0) | 2.2 (1.5,3.0) | 3.7 (2.9,4.6) | 4.3 (3.4,5.4) | 9.1 (7.9,10.6) |
| | Maldives 2016-17 | 67.2 (63.1,71.0) | 17.3 (14.3,20.6) | 3.7 (2.3,5.9) | 1.6 (0.8,2.9) | 3.4 (2.1,5.4) | 4.1 (2.5,6.6) | 2.8 (1.7,4.6) |
| | Tajikistan 2017 | 62.0 (58.4,65.4) | 24.3 (22.0,26.8) | 7.1 (5.2,9.5) | 1.1 (0.7,1.7) | 1.5 (1.1,2.1) | 2.0 (1.5,2.8) | 2.0 (1.4,2.7) |
| South & Southeast Asia | Bangladesh 2014 | 51.4 (48.7,54.1) | 30.5 (28.0,33.0) | 5.4 (4.5,6.6) | 2.9 (2.3,3.7) | 2.6 (2.1,3.4) | 4.1 (3.4,5.1) | 3.0 (2.4,3.8) |
| | Cambodia 2014 | 63.2 (60.7,65.7) | 18.4 (16.7,20.3) | 4.3 (3.4,5.5) | 2.1 (1.5,3.0) | 3.1 (2.3,4.2) | 5.1 (4.1,6.2) | 3.7 (2.9,4.6) |
| | India 2015-16 | 42.0 (41.5,42.6) | 32.8 (32.3,33.3) | 5.5 (5.3,5.7) | 2.1 (2.0,2.3) | 4.9 (4.7,5.1) | 8.1 (7.8,8.4) | 4.5 (4.3,4.8) |
| | Indonesia 2017 | 57.0 (55.4,58.7) | 9.2 (8.4,10.1) | 4.1 (3.6,4.8) | 4.0 (3.4,4.6) | 6.2 (5.6,7.0) | 11.0 (10.1,12.0) | 8.4 (7.6,9.3) |
| | Myanmar 2015-16 | 67.3 (64.2,70.3) | 11.9 (10.1,13.9) | 3.6 (2.7,4.8) | 2.2 (1.5,3.0) | 5.7 (4.5,7.3) | 6.3 (5.1,7.8) | 3.0 (2.2,4.1) |
| | Nepal 2016 | 55.1 (52.1,58.1) | 26.3 (23.9,28.8) | 6.5 (5.4,7.9) | 2.3 (1.8,3.1) | 2.8 (2.0,3.8) | 5.7 (4.3,7.4) | 1.3 (0.8,2.0) |
| | Pakistan 2017-18 | 19.7 (17.5,22.1) | 19.0 (17.1,21.0) | 10.7 (9.2,12.2) | 6.7 (5.7,8.0) | 11.3 (9.8,12.9) | 25.8 (23.2,28.6) | 6.9 (5.7,8.3) |
| | Philippines 2017 | 57.3 (54.7,59.8) | 17.4 (15.6,19.3) | 7.3 (6.0,8.9) | 3.4 (2.7,4.3) | 2.9 (2.3,3.7) | 4.6 (3.2,6.4) | 7.2 (6.1,8.5) |
| | Timor-Leste 2016 | 75.9 (73.3,78.4) | 14.6 (12.7,16.8) | 1.5 (1.1,2.2) | 1.4 (0.9,2.0) | 1.8 (1.2,2.7) | 1.4 (1.0,2.1) | 3.3 (2.5,4.3) |
| Sub-Saharan Africa | Angola 2015-16 | 48.9 (46.3,51.6) | 28.8 (26.6,31.0) | 4.6 (3.8,5.6) | 2.3 (1.8,2.9) | 6.6 (5.5,7.8) | 4.3 (3.6,5.0) | 4.6 (3.9,5.4) |
| | Benin 2017-18 | 54.8 (52.8,56.7) | 19.5 (18.0,21.2) | 8.4 (7.5,9.3) | 3.4 (2.9,4.0) | 6.6 (5.8,7.5) | 4.5 (3.9,5.3) | 2.8 (2.4,3.3) |
| | Burundi 2016-2017 | 85.4 (84.2,86.6) | 10.6 (9.6,11.7) | 1.4 (1.1,1.8) | 0.9 (0.7,1.3) | 0.5 (0.3,0.7) | 0.5 (0.3,0.7) | 0.7 (0.5,1.0) |
| | Chad 2014-15 | 23.2 (21.0,25.6) | 9.0 (7.9,10.2) | 6.5 (5.7,7.5) | 2.7 (2.2,3.3) | 12.1 (10.9,13.4) | 41.8 (39.4,44.1) | 4.7 (4.0,5.6) |
| | Ethiopia 2016 | 74.2 (71.8,76.4) | 12.5 (10.9,14.2) | 3.7 (2.9,4.7) | 2.7 (2.1,3.4) | 1.4 (1.0,1.9) | 3.1 (2.3,4.2) | 2.5 (1.9,3.4) |
| | Ghana 2014 | 56.3 (53.3,59.2) | 20.7 (18.4,23.2) | 6.1 (5.0,7.5) | 5.3 (4.2,6.7) | 4.6 (3.6,5.8) | 5.9 (4.9,7.2) | 1.1 (0.7,1.8) |
| | Kenya 2014 | 62.7 (60.5,64.9) | 16.9 (15.3,18.6) | 6.5 (5.5,7.6) | 5.4 (4.5,6.5) | 3.5 (2.7,4.5) | 3.7 (2.9,4.7) | 1.4 (0.9,2.0) |
| | Lesotho 2014 | 65.7 (62.5,68.7) | 12.5 (10.4,14.9) | 5.6 (4.3,7.1) | 3.3 (2.4,4.5) | 4.8 (3.7,6.3) | 3.1 (2.2,4.3) | 5.1 (3.8,7.0) |
| | Malawi 2015-16 | 77.1 (75.7,78.5) | 14.9 (13.7,16.1) | 3.2 (2.7,3.8) | 2.1 (1.7,2.7) | 0.8 (0.6,1.2) | 0.5 (0.3,0.8) | 1.3 (1.0,1.7) |
| | Senegal 2016 | 33.8 (31.8,35.8) | 27.3 (25.6,29.1) | 17.6 (16.1,19.1) | 12.1 (10.7,13.6) | 3.3 (2.7,4.1) | 3.9 (3.1,4.9) | 2.1 (1.6,2.6) |
| | South Africa 2016 | 68.3 (65.1,71.4) | 8.5 (6.8,10.6) | 2.8 (1.9,4.0) | 1.8 (1.2,2.7) | 1.4 (0.9,2.1) | 2.1 (1.2,3.6) | 15.1 (12.9,17.7) |
| | Tanzania 2015-16 | 51.7 (49.3,54.0) | 25.3 (23.6,27.1) | 9.0 (8.0,10.2) | 7.8 (6.8,9.0) | 3.4 (2.7,4.3) | 2.0 (1.6,2.6) | 0.8 (0.5,1.2) |
| | Uganda 2016 | 66.8 (65.2,68.5) | 20.0 (18.7,21.4) | 5.2 (4.5,5.9) | 2.9 (2.5,3.4) | 1.9 (1.6,2.4) | 1.6 (1.3,2.0) | 1.5 (1.2,2.0) |
| | Zimbabwe 2015 | 58.3 (55.3,61.2) | 22.7 (20.6,25.0) | 7.2 (6.1,8.5) | 6.1 (5.0,7.4) | 2.6 (1.8,3.7) | 1.8 (1.2,2.5) | 1.3 (0.9,1.9) |
| Latin America & Caribbean | Guatemala 2014-15 | 63.5 (61.6,65.4) | 11.9 (10.7,13.2) | 6.3 (5.5,7.1) | 5.3 (4.5,6.1) | 3.6 (3.0,4.2) | 4.2 (3.6,4.9) | 5.3 (4.6,6.2) |
| | Haiti 2016-17 | 47.9 (45.4,50.5) | 24.4 (22.1,26.8) | 8.5 (7.2,10.0) | 3.0 (2.2,3.9) | 5.7 (4.7,7.0) | 5.6 (4.5,6.9) | 5.0 (3.8,6.4) |

[1] For the countries included in the survival analysis, this column represents the proportion of cases censored.

Supplement to: Mallick L, Wang W, Farid S, Pullum T. Initiation of Breastfeeding in Low- and Middle-Income Countries: A Time-to-Event Analysis. *Glob Health Sci Pract*. 2021;9(2). https://doi.org/10.9745/GHSP-D-20-00361

**Supplement Table 2a. Background characteristics of women included in the survival analysis, by country, Europe and Asia**

| | Albania 2017-18 | | Armenia 2015-16 | | Jordan 2017-18 | | Maldives 2016-17 | | Tajikistan 2017 | | Indonesia 2017 | | Nepal 2016 | | Pakistan 2017-18 | | Philippines 2017 | | Timor-Leste 2016 | |
|---|---|---|---|---|---|---|---|---|---|---|---|---|---|---|---|---|---|---|---|---|
| | % | N | % | N | % | N | % | N | % | N | % | N | % | N | % | N | % | N | % | N |
| **Place and mode of delivery** | | | | | | | | | | | | | | | | | | | | |
| Home, vaginal | 1.1 | 11 | 0.7 | 4 | 1.0 | 33 | 4.9 | 51 | 10.5 | 253 | 16.8 | 1,009 | 35.9 | 697 | 29.7 | 1,067 | 17.0 | 581 | 49.2 | 1,340 |
| Facility, vaginal | 68.1 | 654 | 79.7 | 503 | 73.4 | 2,302 | 52.0 | 543 | 83.8 | 2,025 | 65.2 | 3,920 | 54.6 | 1,059 | 45.7 | 1,644 | 68.3 | 2,339 | 47.3 | 1,289 |
| Facility, C-section | 30.8 | 296 | 19.6 | 124 | 25.6 | 802 | 43.1 | 450 | 5.7 | 138 | 18.0 | 1,081 | 9.5 | 185 | 24.7 | 887 | 14.8 | 507 | 3.5 | 94 |
| **Immediate skin-to-skin** | | | | | | | | | | | | | | | | | | | | |
| No | 17.9 | 172 | 20.4 | 129 | 26.5 | 830 | 34.3 | 358 | 11.0 | 265 | 39.3 | 2,363 | 37.0 | 718 | 91.7 | 3,299 | 24.7 | 846 | 36.9 | 1,003 |
| Yes | 82.1 | 788 | 79.6 | 503 | 73.5 | 2,306 | 65.7 | 686 | 89.0 | 2,151 | 60.7 | 3,648 | 63.0 | 1,222 | 8.3 | 300 | 75.3 | 2,582 | 63.1 | 1,719 |
| **PNC in first hour after birth** | | | | | | | | | | | | | | | | | | | | |
| No | 56.5 | 543 | 68.7 | 434 | 81.8 | 2,567 | 57.7 | 602 | 82.3 | 1,988 | 88.9 | 5,345 | 76.5 | 1,483 | 55.5 | 1,998 | 73.5 | 2,520 | 96.6 | 2,629 |
| Yes | 43.5 | 418 | 31.3 | 197 | 18.2 | 570 | 42.3 | 442 | 17.7 | 428 | 11.1 | 665 | 23.5 | 457 | 44.5 | 1,601 | 26.5 | 908 | 3.4 | 94 |
| **Number of ANC visits** | | | | | | | | | | | | | | | | | | | | |
| 0-3 visits | 20.2 | 194 | 3.0 | 19 | 7.2 | 226 | 17.2 | 180 | 34.5 | 832 | 9.6 | 576 | 28.6 | 555 | 48.2 | 1,734 | 13.9 | 478 | 23.0 | 625 |
| 4 or more | 79.8 | 767 | 97.0 | 613 | 92.8 | 2,910 | 82.8 | 864 | 65.5 | 1,584 | 90.4 | 5,435 | 71.4 | 1,385 | 51.8 | 1,865 | 86.1 | 2,950 | 77.0 | 2,097 |
| **Size at birth** | | | | | | | | | | | | | | | | | | | | |
| Normal | 90.5 | 870 | 92.9 | 586 | 80.9 | 2,538 | 83.7 | 874 | 88.2 | 2,131 | 90.5 | 5,437 | 79.8 | 1,548 | 71.3 | 2,566 | 85.8 | 2,940 | 81.8 | 2,226 |
| Small or very small | 4.0 | 38 | 4.7 | 30 | 16.0 | 502 | 13.1 | 136 | 6.9 | 166 | 5.9 | 353 | 13.7 | 266 | 23.2 | 835 | 11.1 | 382 | 9.4 | 256 |
| Large or very large | 5.5 | 53 | 2.5 | 16 | 3.1 | 96 | 3.2 | 34 | 4.9 | 119 | 3.7 | 221 | 6.5 | 126 | 5.5 | 197 | 3.1 | 106 | 8.8 | 241 |
| **Sex of child** | | | | | | | | | | | | | | | | | | | | |
| Male | 49.7 | 477 | 51.0 | 322 | 51.8 | 1,626 | 49.2 | 513 | 51.5 | 1,243 | 51.3 | 3,084 | 53.6 | 1,041 | 49.7 | 1,789 | 51.9 | 1,778 | 51.3 | 1,397 |
| Female | 50.3 | 483 | 49.0 | 309 | 48.2 | 1,510 | 50.8 | 531 | 48.5 | 1,173 | 48.7 | 2,927 | 46.4 | 899 | 50.3 | 1,810 | 48.1 | 1,650 | 48.7 | 1,326 |
| **Parity** | | | | | | | | | | | | | | | | | | | | |
| 1 | 37.8 | 363 | 41.5 | 262 | 24.1 | 756 | 36.6 | 382 | 27.1 | 654 | 32.3 | 1,944 | 40.2 | 781 | 22.9 | 825 | 30.1 | 1,030 | 25.0 | 679 |
| 2-3 | 57.4 | 552 | 54.9 | 347 | 40.9 | 1,284 | 53.5 | 559 | 54.4 | 1,314 | 55.0 | 3,304 | 44.5 | 863 | 40.1 | 1,443 | 46.6 | 1,596 | 36.3 | 988 |
| 4+ | 4.8 | 46 | 3.5 | 22 | 35.0 | 1,096 | 9.9 | 103 | 18.6 | 448 | 12.7 | 763 | 15.3 | 297 | 37.0 | 1,331 | 23.4 | 801 | 38.8 | 1,056 |
| **Marital status** | | | | | | | | | | | | | | | | | | | | |
| Not married[1] | 1.2 | 11 | 1.2 | 8 | 0.7 | 23 | 1.8 | 18 | 2.0 | 49 | 1.8 | 111 | 0.3 | 5 | 0.9 | 34 | 7.3 | 250 | 2.1 | 56 |
| Married | 98.8 | 950 | 98.8 | 624 | 99.3 | 3,114 | 98.2 | 1,026 | 98.0 | 2,367 | 98.2 | 5,900 | 99.7 | 1,935 | 99.1 | 3,565 | 92.7 | 3,177 | 97.9 | 2,666 |
| **Exposed to TV, radio, or newspaper** | | | | | | | | | | | | | | | | | | | | |
| Less than once a week | 9.5 | 91 | 8.1 | 51 | 19.9 | 625 | 6.7 | 70 | 14.8 | 358 | 14.8 | 889 | 46.1 | 894 | 51.5 | 1,854 | 19.0 | 652 | 59.9 | 1,631 |
| At least once a week | 90.5 | 870 | 91.9 | 580 | 80.1 | 2,511 | 93.3 | 974 | 85.2 | 2,058 | 85.2 | 5,122 | 53.9 | 1,046 | 48.5 | 1,745 | 81.0 | 2,776 | 40.1 | 1,092 |
| **Employment** | | | | | | | | | | | | | | | | | | | | |
| Not employed | 67.8 | 652 | 77.0 | 486 | 88.7 | 2,781 | 59.3 | 619 | 85.7 | 2,070 | 56.1 | 3,374 | 46.5 | 903 | 84.2 | 3,030 | 60.5 | 2,075 | 66.1 | 1,800 |
| Employed | 32.2 | 309 | 23.0 | 146 | 11.3 | 356 | 40.7 | 425 | 14.3 | 346 | 43.9 | 2,636 | 53.5 | 1,037 | 15.8 | 568 | 39.5 | 1,353 | 33.9 | 923 |
| **Education** | | | | | | | | | | | | | | | | | | | | |
| None or primary | 44.8 | 430 | 5.1 | 32 | 7.3 | 228 | 15.6 | 163 | 7.8 | 188 | 24.1 | 1,447 | 48.4 | 938 | 63.7 | 2,293 | 17.1 | 587 | 39.7 | 1,082 |
| Secondary or higher | 55.2 | 531 | 94.9 | 599 | 92.7 | 2,909 | 84.4 | 881 | 92.2 | 2,228 | 75.9 | 4,563 | 51.6 | 1,002 | 36.3 | 1,306 | 82.9 | 2,841 | 60.3 | 1,640 |
| **Wealth** | | | | | | | | | | | | | | | | | | | | |
| First | 21.7 | 208 | 17.5 | 111 | 25.6 | 804 | 18.7 | 196 | 17.8 | 430 | 20.3 | 1,222 | 21.2 | 411 | 22.1 | 794 | 27.2 | 932 | 19.7 | 537 |
| Second | 19.1 | 183 | 21.5 | 136 | 24.4 | 766 | 22.1 | 231 | 20.1 | 486 | 20.4 | 1,225 | 21.0 | 408 | 19.2 | 692 | 22.0 | 753 | 20.1 | 547 |
| Middle | 19.9 | 191 | 18.2 | 115 | 23.7 | 744 | 21.5 | 224 | 23.3 | 563 | 19.8 | 1,192 | 22.9 | 444 | 22.0 | 790 | 20.2 | 692 | 20.7 | 562 |
| Fourth | 21.5 | 207 | 18.5 | 117 | 16.3 | 510 | 17.7 | 185 | 22.5 | 545 | 20.4 | 1,226 | 20.6 | 399 | 18.5 | 665 | 16.7 | 571 | 20.6 | 560 |
| Highest | 17.8 | 171 | 24.3 | 153 | 10.0 | 313 | 20.0 | 208 | 16.2 | 392 | 19.1 | 1,145 | 14.3 | 278 | 18.3 | 659 | 14.0 | 480 | 19.0 | 516 |
| **Place of residence** | | | | | | | | | | | | | | | | | | | | |
| Urban | 57.3 | 551 | 58.3 | 368 | 88.2 | 2,765 | na | | 19.4 | 468 | 48.2 | 2,896 | 53.6 | 1,041 | 32.9 | 1,185 | 44.0 | 1,507 | 27.3 | 744 |
| Rural | 42.7 | 410 | 41.7 | 264 | 11.8 | 371 | na | | 80.6 | 1,948 | 51.8 | 3,115 | 46.4 | 900 | 67.1 | 2,413 | 56.0 | 1,921 | 72.7 | 1,979 |
| **Total** | | 961 | | 631 | | 3,137 | | 1,044 | | 2,416 | | 6,011 | | 1,940 | | 3,599 | | 3,428 | | 2,723 |

Supplement to: Mallick L, Wang W, Farid S, Pullum T. Initiation of Breastfeeding in Low- and Middle-Income Countries: A Time-to-Event Analysis. *Glob Health Sci Pract*. 2021;9(2).
**https://doi.org/10.9745/GHSP-D-20-00361**

**Supplement Table 2b. Background characteristics of women included in the survival analysis, by country, sub-Saharan Africa and Haiti**

| | Angola 2015-16 | | Benin 2017-18 | | Burundi 2016-2017 | | Ethiopia 2016 | | Malawi 2015-16 | | Senegal 2016 | | South Africa 2016 | | Tanzania 2015-16 | | Uganda 2016 | | Zimbabwe 2015 | | Haiti 2016-17 | |
|---|---|---|---|---|---|---|---|---|---|---|---|---|---|---|---|---|---|---|---|---|---|---|
| | % | N | % | N | % | N | % | N | % | N | % | N | % | N | % | N | % | N | % | N | % | N |
| **Place and mode of delivery** | | | | | | | | | | | | | | | | | | | | | | |
| Home, vaginal | 52.1 | 2,632 | 14.8 | 780 | 14.6 | 779 | 63.9 | 2,641 | 7.0 | 453 | 19.9 | 860 | 3.9 | 45 | 35.1 | 1,429 | 23.5 | 1,340 | 18.9 | 451 | 63.0 | 1,430 |
| Facility, vaginal | 44.0 | 2,224 | 80.3 | 4,221 | 80.3 | 4,278 | 33.7 | 1,394 | 86.5 | 5,615 | 74.9 | 3,234 | 72.9 | 844 | 58.6 | 2,386 | 69.5 | 3,966 | 75.2 | 1797 | 32.4 | 736 |
| Facility, C-section | 3.9 | 199 | 4.8 | 253 | 5.1 | 274 | 2.4 | 101 | 6.5 | 425 | 5.2 | 224 | 23.3 | 269 | 6.4 | 259 | 7.1 | 403 | 6.0 | 142 | 4.6 | 104 |
| **Immediate skin-to-skin** | | | | | | | | | | | | | | | | | | | | | | |
| No | 53.2 | 2,691 | 23.7 | 1,245 | 86.7 | 4,622 | 72.0 | 2,977 | 34.4 | 2,236 | 47.8 | 2,066 | 34.1 | 395 | 66.0 | 2,690 | 25.7 | 1,465 | 49.4 | 1180 | 70.2 | 1,595 |
| Yes | 46.8 | 2,364 | 76.3 | 4,008 | 13.3 | 708 | 28.0 | 1,159 | 65.6 | 4,256 | 52.2 | 2,253 | 65.9 | 763 | 34.0 | 1,384 | 74.3 | 4,243 | 50.6 | 1210 | 29.8 | 676 |
| **PNC in first hour after birth** | | | | | | | | | | | | | | | | | | | | | | |
| No | 95.5 | 4,826 | 73.4 | 3,856 | 78.7 | 4,195 | 95.4 | 3,944 | 77.3 | 5,022 | 44.3 | 1,914 | 46.6 | 540 | 90.3 | 3,679 | 78.7 | 4,492 | 67.0 | 1602 | 85.1 | 1,932 |
| Yes | 4.5 | 230 | 26.6 | 1,398 | 21.3 | 1,135 | 4.6 | 192 | 22.7 | 1,471 | 55.7 | 2,404 | 53.4 | 618 | 9.7 | 395 | 21.3 | 1,216 | 33.0 | 788 | 14.9 | 339 |
| **Number of ANC visits** | | | | | | | | | | | | | | | | | | | | | | |
| 0-3 visits | 38.9 | 1,968 | 49.3 | 2,591 | 48.2 | 2,570 | 66.4 | 2,748 | 51.6 | 3,353 | 44.4 | 1,916 | 24.6 | 285 | 51.9 | 2,115 | 39.5 | 2,252 | 26.3 | 629 | 37.1 | 843 |
| 4 or more | 61.1 | 3,088 | 50.7 | 2,663 | 51.8 | 2,760 | 33.6 | 1,388 | 48.4 | 3,139 | 55.6 | 2,403 | 75.4 | 873 | 48.1 | 1,959 | 60.5 | 3,456 | 73.7 | 1761 | 62.9 | 1,428 |
| **Size at birth** | | | | | | | | | | | | | | | | | | | | | | |
| Normal | 74.6 | 3,769 | 77.2 | 4,058 | 80.4 | 4,285 | 48.3 | 1,998 | 76.5 | 4,967 | 71.0 | 3,066 | 78.3 | 907 | 82.3 | 3,355 | 73.6 | 4,203 | 81.9 | 1958 | 58.5 | 1,329 |
| Small or very small | 10.1 | 509 | 12.2 | 643 | 11.3 | 604 | 27.4 | 1,132 | 12.0 | 781 | 18.1 | 780 | 16.5 | 191 | 9.1 | 370 | 12.5 | 714 | 9.8 | 235 | 26.7 | 605 |
| Large or very large | 15.4 | 778 | 10.5 | 553 | 8.3 | 441 | 24.3 | 1,005 | 11.5 | 745 | 11.0 | 473 | 5.2 | 60 | 8.6 | 349 | 13.9 | 791 | 8.3 | 197 | 14.8 | 337 |
| **Sex of child** | | | | | | | | | | | | | | | | | | | | | | |
| Male | 49.6 | 2,509 | 50.0 | 2,629 | 50.6 | 2,697 | 48.0 | 1,986 | 50.6 | 3,286 | 50.5 | 2,182 | 51.7 | 598 | 50.9 | 2,074 | 51.1 | 2,915 | 50.4 | 1204 | 49.1 | 1,115 |
| Female | 50.4 | 2,547 | 50.0 | 2,625 | 49.4 | 2,633 | 52.0 | 2,150 | 49.4 | 3,206 | 49.5 | 2,137 | 48.3 | 560 | 49.1 | 2,000 | 48.9 | 2,793 | 49.6 | 1186 | 50.9 | 1,156 |
| **Parity** | | | | | | | | | | | | | | | | | | | | | | |
| 1 | 20.8 | 1,051 | 21.8 | 1,147 | 17.3 | 921 | 20.5 | 847 | 27.5 | 1,787 | 24.6 | 1,061 | 34.8 | 403 | 27.1 | 1,105 | 22.4 | 1,279 | 26.5 | 634 | 28.9 | 656 |
| 2-3 | 35.4 | 1,789 | 35.9 | 1,885 | 34.5 | 1,839 | 30.5 | 1,262 | 36.3 | 2,357 | 34.6 | 1,493 | 51.6 | 597 | 34.3 | 1,397 | 34.8 | 1,986 | 44.8 | 1071 | 40.1 | 911 |
| 4+ | 43.8 | 2,216 | 42.3 | 2,222 | 48.2 | 2,570 | 49.0 | 2,027 | 36.2 | 2,348 | 40.9 | 1,765 | 13.6 | 158 | 38.6 | 1,573 | 42.8 | 2,443 | 28.7 | 685 | 31.0 | 704 |
| **Marital status** | | | | | | | | | | | | | | | | | | | | | | |
| Not married[1] | 25.4 | 1,282 | 5.8 | 303 | 8.9 | 475 | 4.4 | 182 | 16.3 | 1,061 | 6.3 | 272 | 57.0 | 660 | 18.4 | 750 | 15.8 | 902 | 13.4 | 320 | 13.9 | 317 |
| Married | 74.6 | 3,773 | 94.2 | 4,950 | 91.1 | 4,855 | 95.6 | 3,954 | 83.7 | 5,431 | 93.7 | 4,047 | 43.0 | 498 | 81.6 | 3,324 | 84.2 | 4,806 | 86.6 | 2071 | 86.1 | 1,954 |
| **Exposed to TV, radio, or newspaper** | | | | | | | | | | | | | | | | | | | | | | |
| Less than once a week | 58.2 | 2,941 | 60.1 | 3,155 | 72.7 | 3,876 | 80.6 | 3,334 | 65.9 | 4,281 | 28.3 | 1,221 | 20.8 | 240 | 52.9 | 2,156 | 37.8 | 2,160 | 50.2 | 1201 | 66.4 | 1,508 |
| At least once a week | 41.8 | 2,115 | 39.9 | 2,099 | 27.3 | 1,455 | 19.4 | 802 | 34.1 | 2,212 | 71.7 | 3,098 | 79.2 | 918 | 47.1 | 1,918 | 62.2 | 3,548 | 49.8 | 1189 | 33.6 | 763 |
| **Employment** | | | | | | | | | | | | | | | | | | | | | | |
| Not employed | 28.9 | 1,460 | 20.1 | 1,057 | 7.0 | 373 | 58.2 | 2,406 | 31.8 | 2,065 | 44.0 | 1,899 | 70.7 | 819 | 19.2 | 782 | 18.7 | 1,066 | 53.7 | 1284 | 40.9 | 928 |
| Employed | 71.1 | 3,596 | 79.9 | 4,197 | 93.0 | 4,957 | 41.8 | 1,730 | 68.2 | 4,428 | 56.0 | 2,420 | 29.3 | 339 | 80.8 | 3,292 | 81.3 | 4,642 | 46.3 | 1106 | 59.1 | 1,343 |
| **Education** | | | | | | | | | | | | | | | | | | | | | | |
| None or primary | 66.5 | 3,362 | 81.8 | 4,295 | 88.0 | 4,693 | 91.2 | 3,773 | 78.7 | 5,109 | 81.3 | 3,509 | 9.7 | 112 | 83.1 | 3,384 | 70.4 | 4,017 | 33.2 | 793 | 57.7 | 1,311 |
| Secondary or higher | 33.5 | 1,693 | 18.2 | 958 | 12.0 | 638 | 8.8 | 363 | 21.3 | 1,383 | 18.7 | 810 | 90.3 | 1046 | 16.9 | 691 | 29.6 | 1,690 | 66.8 | 1597 | 42.3 | 960 |
| **Wealth** | | | | | | | | | | | | | | | | | | | | | | |
| First | 21.8 | 1,100 | 21.0 | 1,102 | 22.1 | 1,176 | 23.2 | 961 | 25.4 | 1,651 | 24.5 | 1,060 | 23.5 | 272 | 24.6 | 1,003 | 22.4 | 1,281 | 24.8 | 593 | 26.5 | 602 |
| Second | 23.3 | 1,178 | 20.7 | 1,090 | 22.1 | 1,179 | 22.2 | 920 | 22.6 | 1,469 | 22.3 | 963 | 23.6 | 273 | 20.9 | 853 | 21.3 | 1,215 | 20.5 | 490 | 22.7 | 516 |
| Middle | 21.8 | 1,103 | 20.7 | 1,086 | 20.6 | 1,099 | 20.8 | 859 | 19.3 | 1,254 | 20.6 | 888 | 20.0 | 232 | 18.6 | 758 | 19.1 | 1,089 | 18.2 | 434 | 21.7 | 493 |
| Fourth | 18.0 | 909 | 20.0 | 1,048 | 18.9 | 1,008 | 18.3 | 758 | 16.9 | 1,097 | 17.2 | 744 | 18.7 | 216 | 19.0 | 776 | 17.6 | 1,005 | 22.5 | 538 | 15.8 | 358 |
| Highest | 15.2 | 766 | 17.6 | 927 | 16.3 | 869 | 15.4 | 638 | 15.7 | 1,022 | 15.4 | 664 | 14.2 | 164 | 16.8 | 684 | 19.6 | 1,119 | 14.0 | 335 | 13.3 | 302 |
| **Place of residence** | | | | | | | | | | | | | | | | | | | | | | |
| Urban | 60.8 | 3,075 | 38.2 | 2,009 | 9.0 | 478 | 11.8 | 489 | 13.7 | 886 | 36.0 | 1,554 | 63.2 | 731 | 27.4 | 1,115 | 21.2 | 1,207 | 27.9 | 666 | 32.9 | 746 |
| Rural | 39.2 | 1,980 | 61.8 | 3,245 | 91.0 | 4,852 | 88.2 | 3,647 | 86.3 | 5,606 | 64.0 | 2,765 | 36.8 | 427 | 72.6 | 2,959 | 78.8 | 4,501 | 72.1 | 1724 | 67.1 | 1,525 |
| **Total** | | 5,056 | | 5,254 | | 5,330 | | 4,136 | | 6,492 | | 4,319 | | 1158 | | 4,074 | | 5,708 | | 2390 | | 2,271 |

[1]Sample of women is ever-married only in Jordan and Pakistan; region not shown.

Supplement to: Mallick L, Wang W, Farid S, Pullum T. Initiation of Breastfeeding in Low- and Middle-Income Countries: A Time-to-Event Analysis. *Glob Health Sci Pract*. 2021;9(2). https://doi.org/10.9745/GHSP-D-20-00361

**Supplement Table 3a. Time ratio (TR) and 95% confidence intervals (CI) according to multivariable log-logistic regressions of time to initiation of breastfeeding, Europe and Asia**

| | Albania 2017-18 | | Armenia 2015-16 | | Jordan 2017-18 | | Maldives 2016-17 | | Tajikistan 2017 | |
|---|---|---|---|---|---|---|---|---|---|---|
| | TR | 95% CI | TR | 95% CI | TR | 95% CI | TR | 95% CI | TR | 95% CI |
| **Place and mode of delivery (ref = Facility, vaginal)** | | | | | | | | | | |
| Home, vaginal | 0.9 | 0.7, 1.2 | 0.9 | 0.4, 1.8 | 1.1 | 0.8, 1.6 | 1.3 | 0.9, 2.0 | 0.8** | 0.7, 0.9 |
| Facility, C-section | 1.3*** | 1.1, 1.5 | 3.0*** | 1.9, 4.7 | 1.6*** | 1.4, 1.8 | 1.1 | 1.0, 1.3 | 2.7*** | 1.7, 4.1 |
| **Immediate skin-to-skin (ref = no)** | | | | | | | | | | |
| Yes | 0.2*** | 0.2, 0.3 | 0.6** | 0.4, 0.8 | 0.2*** | 0.2, 0.3 | 0.7** | 0.6, 0.9 | 0.5*** | 0.4, 0.7 |
| **PNC in first hour after birth (ref = no)** | | | | | | | | | | |
| Yes | 0.7*** | 0.6, 0.8 | 1.3* | 1.0, 1.7 | 1.2* | 1.0, 1.4 | 1.3* | 1.1, 1.6 | 0.7*** | 0.7, 0.8 |
| **Number of ANC visits (ref = 4 or more)** | | | | | | | | | | |
| 0-3 visits[1] | 1.0 | 0.9, 1.3 | 0.7 | 0.4, 1.1 | 0.9 | 0.8, 1.1 | 1.2 | 0.9, 1.5 | 1.0 | 0.9, 1.1 |
| **Size at birth (ref = normal)** | | | | | | | | | | |
| Small or very small | 1.0 | 0.8, 1.4 | 1.1 | 0.5, 2.0 | 0.9* | 0.8, 1.0 | 1.5* | 1.0, 2.2 | 1.2 | 1.0, 1.5 |
| Large or very large | 0.8 | 0.7, 1.0 | 1.0 | 0.5, 2.1 | 1.1 | 0.9, 1.5 | 1.2 | 0.5, 2.8 | 1.0 | 0.9, 1.2 |
| **Sex of child (ref = male)** | | | | | | | | | | |
| Female | 1.0 | 0.8, 1.1 | 0.9 | 0.7, 1.2 | 1.0 | 0.9, 1.1 | 0.9 | 0.8, 1.1 | 1.0 | 0.9, 1.1 |
| **Parity (ref = 1)** | | | | | | | | | | |
| 2-3 | 0.9 | 0.8, 1.0 | 0.8* | 0.6, 1.0 | 0.9 | 0.8, 1.1 | 0.8 | 0.6, 1.0 | 1.0 | 0.9, 1.1 |
| 4+ | 1.1 | 0.8, 1.5 | 0.5* | 0.3, 1.0 | 1.0 | 0.9, 1.1 | 0.7 | 0.5, 1.0 | 1.0 | 0.8, 1.1 |
| **Marital status** | | | | | | | | | | |
| Not married[1] | 0.9 | 0.8, 1.2 | 0.3* | 0.1, 1.0 | 1.3 | 0.4, 4.3 | 1.9 | 0.5, 7.0 | 1.1 | 0.8, 1.5 |
| **Exposed to TV, radio, or newspaper (ref = < once per week** | | | | | | | | | | |
| At least once a week | 0.8 | 0.6, 1.1 | 0.6* | 0.4, 1.0 | 0.9 | 0.8, 1.0 | 0.9 | 0.7, 1.2 | 1.0 | 0.9, 1.1 |
| **Employment (ref = not employed)** | | | | | | | | | | |
| Employed | 1.2* | 1.0, 1.4 | 1.0 | 0.7, 1.2 | 1.0 | 0.9, 1.1 | 1.0 | 0.8, 1.2 | 0.9 | 0.8, 1.0 |
| **Education (ref = none or primary)** | | | | | | | | | | |
| Secondary or higher | 1.1 | 0.9, 1.3 | 1.1 | 0.6, 1.9 | 1.0 | 0.8, 1.1 | 1.0 | 0.7, 1.3 | 1.1 | 1.0, 1.3 |
| **Wealth (ref = Lowest)** | | | | | | | | | | |
| Second | 1.0 | 0.8, 1.3 | 1.1 | 0.8, 1.5 | 1.0 | 0.9, 1.1 | 1.1 | 0.9, 1.2 | 1.1 | 0.9, 1.3 |
| Middle | 0.9 | 0.7, 1.1 | 1.2 | 0.9, 1.7 | 1.1 | 0.9, 1.2 | 0.9* | 0.7, 1.0 | 1.1 | 1.0, 1.3 |
| Fourth | 0.8 | 0.6, 1.1 | 0.9 | 0.6, 1.4 | 1.0 | 0.8, 1.1 | 0.8* | 0.6, 1.0 | 1.1 | 0.9, 1.2 |
| Highest | 0.7* | 0.5, 1.0 | 0.9 | 0.6, 1.4 | 0.9 | 0.7, 1.1 | 0.9 | 0.5, 1.5 | 1.1 | 0.8, 1.4 |
| **Place of residence (ref = urban)** | | | | | | | | | | |
| Rural | 0.9 | 0.8, 1.1 | 0.7* | 0.5, 1.0 | 1.0 | 0.9, 1.1 | na | na | 0.9 | 0.8, 1.1 |

Supplement to: Mallick L, Wang W, Farid S, Pullum T. Initiation of Breastfeeding in Low- and Middle-Income Countries: A Time-to-Event Analysis. *Glob Health Sci Pract*. 2021;9(2).
**https://doi.org/10.9745/GHSP-D-20-00361**

## Supplement Table 3a—*Continued*

| | Indonesia 2017 | | Nepal 2016 | | Pakistan 2017-18 | | Philippines 2017 | | Timor-Leste 2016 | |
|---|---|---|---|---|---|---|---|---|---|---|
| | **TR** | **95% CI** | **TR** | **95% CI** | **TR** | **95% CI** | **TR** | **95% CI** | **TR** | **95% CI** |
| **Place and mode of delivery (ref = Facility, vaginal)** | | | | | | | | | | |
| Home, vaginal | 0.9* | 0.7, 1.0 | 1.3** | 1.1, 1.5 | 1.0 | 0.8, 1.3 | 1.0 | 0.8, 1.2 | 1.0 | 1.0, 1.1 |
| Facility, C-section | 2.4*** | 1.9, 2.9 | 2.9*** | 2.2, 3.9 | 3.5*** | 2.9, 4.3 | 1.9** | 1.3, 3.0 | 1.6* | 1.0, 2.5 |
| **Immediate skin-to-skin (ref = no)** | | | | | | | | | | |
| Yes | 0.5*** | 0.4, 0.6 | 0.6*** | 0.6, 0.7 | 0.7** | 0.5, 0.9 | 0.5*** | 0.4, 0.7 | 1.0 | 1.0, 1.1 |
| **PNC in first hour after birth (ref = no)** | | | | | | | | | | |
| Yes | 0.9 | 0.8, 1.1 | 0.8** | 0.7, 0.9 | 0.9 | 0.7, 1.0 | 0.8*** | 0.7, 0.9 | 0.9 | 0.8, 1.1 |
| **Number of ANC visits (ref = 4 or more)** | | | | | | | | | | |
| 0-3 visits[1] | 1.0 | 0.8, 1.1 | 1.0 | 0.9, 1.1 | 1.3* | 1.0, 1.5 | 0.9 | 0.8, 1.1 | 1.0 | 0.9, 1.1 |
| **Size at birth (ref = normal)** | | | | | | | | | | |
| Small or very small | 1.1 | 0.9, 1.4 | 1.1 | 0.9, 1.4 | 1.6*** | 1.3, 1.9 | 1.2 | 1.0, 1.4 | 1.1 | 1.0, 1.2 |
| Large or very large | 1.2 | 0.9, 1.7 | 0.9 | 0.7, 1.1 | 0.8 | 0.6, 1.1 | 1.3 | 0.9, 1.9 | 1.0 | 0.9, 1.1 |
| **Sex of child (ref = male)** | | | | | | | | | | |
| Female | 1.0 | 0.9, 1.1 | 0.9 | 0.8, 1.0 | 1.1 | 0.9, 1.3 | 1.0 | 0.9, 1.1 | 1.0 | 0.9, 1.0 |
| **Parity (ref = 1)** | | | | | | | | | | |
| 2-3 | 0.8*** | 0.7, 0.9 | 0.8* | 0.7, 1.0 | 0.8** | 0.6, 0.9 | 0.8* | 0.7, 1.0 | 0.9** | 0.8, 1.0 |
| 4+ | 0.7*** | 0.6, 0.8 | 0.8* | 0.7, 1.0 | 0.7*** | 0.5, 0.8 | 0.8* | 0.6, 1.0 | 0.9*** | 0.8, 0.9 |
| **Marital status** | | | | | | | | | | |
| Not married[1] | 0.8 | 0.6, 1.1 | 1.7 | 0.9, 3.2 | 1.4 | 0.7, 2.5 | 1.2 | 0.9, 1.5 | 1.0 | 0.8, 1.1 |
| **Exposed to TV, radio, or newspaper (ref = < once per week** | | | | | | | | | | |
| At least once a week | 1.1 | 1.0, 1.3 | 1.0 | 0.9, 1.2 | 0.8 | 0.7, 1.0 | 1.0 | 0.9, 1.1 | 1.0 | 0.9, 1.1 |
| **Employment (ref = not employed)** | | | | | | | | | | |
| Employed | 1.0 | 0.9, 1.1 | 0.9 | 0.8, 1.1 | 1.1 | 0.9, 1.4 | 1.0 | 0.9, 1.1 | 1.0 | 0.9, 1.0 |
| **Education (ref = none or primary)** | | | | | | | | | | |
| Secondary or higher | 0.9 | 0.8, 1.0 | 0.8* | 0.7, 1.0 | 1.0 | 0.8, 1.3 | 1.1 | 1.0, 1.3 | 1.0 | 1.0, 1.1 |
| **Wealth (ref = Lowest)** | | | | | | | | | | |
| Second | 1.1 | 0.9, 1.2 | 1.2* | 1.0, 1.4 | 0.9 | 0.7, 1.1 | 1.1 | 1.0, 1.2 | 0.9** | 0.8, 1.0 |
| Middle | 1.1 | 0.9, 1.3 | 1.2 | 1.0, 1.4 | 0.8 | 0.6, 1.2 | 1.0 | 0.9, 1.2 | 0.9 | 0.9, 1.0 |
| Fourth | 1.2* | 1.0, 1.5 | 1.2 | 1.0, 1.4 | 0.8 | 0.5, 1.2 | 1.4* | 1.1, 1.8 | 1.0 | 0.9, 1.1 |
| Highest | 1.1 | 0.9, 1.3 | 1.2 | 1.0, 1.5 | 0.7 | 0.4, 1.1 | 1.0 | 0.7, 1.3 | 0.9* | 0.8, 1.0 |
| **Place of residence (ref = urban)** | | | | | | | | | | |
| Rural | 1.1 | 0.9, 1.2 | 1.1 | 1.0, 1.3 | 1.1 | 0.8, 1.4 | 0.9 | 0.8, 1.1 | 1.0 | 0.9, 1.1 |

[1]Sample of women is ever-married only in Jordan and Pakistan; *P<0.05, **P<0.01, ***P<0.001; region not shown.

Supplement to: Mallick L, Wang W, Farid S, Pullum T. Initiation of Breastfeeding in Low- and Middle-Income Countries: A Time-to-Event Analysis. *Glob Health Sci Pract*. 2021;9(2). https://doi.org/10.9745/GHSP-D-20-00361

**Supplement Table 3b. Time ratio (TR) and 95% confidence intervals (CI) according to multivariable log-logistic regressions of time to initiation of breastfeeding, sub-Saharan Africa and Haiti**

| | Angola 2015-16 | | Benin 2017-18 | | Burundi 2016-2017 | | Ethiopia 2016 | | Malawi 2015-16 | |
|---|---|---|---|---|---|---|---|---|---|---|
| | **TR** | **95% CI** | **TR** | **95% CI** | **TR** | **95% CI** | **TR** | **95% CI** | **TR** | **95% CI** |
| **Place and mode of delivery (ref = Facility, vaginal)** | | | | | | | | | | |
| Home, vaginal | 1.0 | 0.9, 1.1 | 1.0 | 0.9, 1.2 | 1.1*** | 1.0, 1.1 | 1.0 | 1.0, 1.1 | 1.1* | 1.0, 1.2 |
| Facility, C-section | 6.6*** | 3.0, 14.7 | 4.2*** | 3.0, 6.0 | 2.8*** | 2.5, 3.3 | 2.5** | 1.3, 4.8 | 1.5*** | 1.3, 1.8 |
| **Immediate skin-to-skin (ref = no)** | | | | | | | | | | |
| Yes | 0.7*** | 0.7, 0.9 | 0.9* | 0.7, 1.0 | 1.0* | 1.0, 1.0 | 0.9*** | 0.8, 0.9 | 0.9*** | 0.9, 0.9 |
| **PNC in first hour after birth (ref = no)** | | | | | | | | | | |
| Yes | 0.9 | 0.7, 1.2 | 0.9* | 0.8, 1.0 | 1.0 | 1.0, 1.0 | 1.1 | 0.9, 1.3 | 1.0 | 0.9, 1.0 |
| **Number of ANC visits (ref = 4 or more)** | | | | | | | | | | |
| 0-3 visits[1] | 1.1 | 1.0, 1.2 | 0.9 | 0.9, 1.0 | 1.0 | 1.0, 1.0 | 1.0 | 0.9, 1.1 | 1.0 | 1.0, 1.0 |
| **Size at birth (ref = normal)** | | | | | | | | | | |
| Small or very small | 1.1 | 0.9, 1.3 | 1.0 | 0.9, 1.1 | 1.0 | 1.0, 1.0 | 1.1* | 1.0, 1.2 | 1.0 | 1.0, 1.1 |
| Large or very large | 0.9 | 0.8, 1.0 | 0.9 | 0.8, 1.0 | 1.0 | 1.0, 1.0 | 1.0 | 1.0, 1.1 | 1.0 | 0.9, 1.0 |
| **Sex of child (ref = male)** | | | | | | | | | | |
| Female | 1.0 | 1.0, 1.1 | 1.0 | 0.9, 1.1 | 1.0 | 1.0, 1.0 | 1.0 | 0.9, 1.0 | 1.0 | 1.0, 1.1 |
| **Parity (ref = 1)** | | | | | | | | | | |
| 2-3 | 1.0 | 0.9, 1.2 | 0.8*** | 0.7, 0.9 | 1.0 | 1.0, 1.0 | 0.9** | 0.8, 1.0 | 0.9** | 0.9, 1.0 |
| 4+ | 1.0 | 0.8, 1.1 | 0.8*** | 0.7, 0.9 | 1.0 | 1.0, 1.0 | 0.9* | 0.8, 1.0 | 1.0 | 0.9, 1.0 |
| **Marital status** | | | | | | | | | | |
| Not married | 1.0 | 0.9, 1.1 | 1.0 | 0.8, 1.2 | 1.0 | 1.0, 1.0 | 1.0 | 0.9, 1.2 | 1.0 | 0.9, 1.0 |
| **Exposed to TV, radio, or newspaper (ref = < once per week** | | | | | | | | | | |
| At least once a week | 1.2** | 1.1, 1.3 | 1.0 | 0.9, 1.1 | 1.0 | 1.0, 1.0 | 1.0 | 0.9, 1.1 | 1.0 | 0.9, 1.0 |
| **Employment (ref = not employed)** | | | | | | | | | | |
| Employed | 1.1 | 1.0, 1.2 | 1.0 | 0.9, 1.1 | 1.0 | 0.9, 1.0 | 1.0 | 0.9, 1.1 | 1.0* | 1.0, 1.1 |
| **Education (ref = none or primary)** | | | | | | | | | | |
| Secondary or higher | 1.1 | 1.0, 1.2 | 1.1 | 1.0, 1.2 | 1.0 | 1.0, 1.0 | 1.0 | 0.9, 1.2 | 1.0 | 1.0, 1.1 |
| **Wealth (ref = Lowest)** | | | | | | | | | | |
| Second | 1.0 | 0.9, 1.2 | 0.9 | 0.8, 1.1 | 1.0 | 1.0, 1.0 | 1.0 | 0.9, 1.1 | 1.0 | 1.0, 1.0 |
| Middle | 1.0 | 0.8, 1.2 | 1.0 | 0.8, 1.1 | 1.0 | 1.0, 1.0 | 1.0 | 0.9, 1.1 | 1.0 | 1.0, 1.0 |
| Fourth | 1.2 | 0.9, 1.5 | 1.0 | 0.8, 1.1 | 1.0 | 1.0, 1.0 | 1.1 | 1.0, 1.2 | 1.0 | 1.0, 1.1 |
| Highest | 1.1 | 0.9, 1.3 | 0.9 | 0.8, 1.1 | 1.0 | 1.0, 1.0 | 1.0 | 0.9, 1.2 | 1.0 | 1.0, 1.1 |
| **Place of residence (ref = urban)** | | | | | | | | | | |
| Rural | 1.2 | 1.0, 1.4 | 1.0 | 0.9, 1.1 | 1.0 | 0.9, 1.0 | 1.1 | 0.9, 1.3 | 0.8*** | 0.8, 0.9 |

Supplement to: Mallick L, Wang W, Farid S, Pullum T. Initiation of Breastfeeding in Low- and Middle-Income Countries: A Time-to-Event Analysis. *Glob Health Sci Pract*. 2021;9(2).
**https://doi.org/10.9745/GHSP-D-20-00361**

**Supplement Table 3b—*Continued***

| | Senegal 2016 | | South Africa 2016 | | Tanzania 2015-16 | | Uganda 2016 | | Zimbabwe 2015 | | Haiti 2016-17 | |
|---|---|---|---|---|---|---|---|---|---|---|---|---|
| | **TR** | **95% CI** | **TR** | **95% CI** | **TR** | **95% CI** | **TR** | **95% CI** | **TR** | **95% CI** | **TR** | **95% CI** |
| **Place and mode of delivery (ref = Facility, vaginal)** | | | | | | | | | | | | |
| Home, vaginal | 1.4*** | 1.2, 1.6 | 1.0 | 0.8, 1.2 | 1.5*** | 1.3, 1.6 | 1.1* | 1.0, 1.1 | 1.6*** | 1.3, 1.9 | 0.9 | 0.8, 1.1 |
| Facility, C-section | 9.3*** | 6.6, 13.2 | 1.0 | 1.0, 1.1 | 4.9*** | 3.9, 6.1 | 2.4*** | 1.9, 2.9 | 4.5*** | 3.3, 6.1 | 3.4*** | 1.8, 6.3 |
| **Immediate skin-to-skin (ref = no)** | | | | | | | | | | | | |
| Yes | 0.7*** | 0.6, 0.7 | 0.8** | 0.8, 0.9 | 0.8*** | 0.8, 0.9 | 0.8*** | 0.8, 0.9 | 0.9** | 0.8, 1.0 | 0.6*** | 0.5, 0.8 |
| **PNC in first hour after birth (ref = no)** | | | | | | | | | | | | |
| Yes | 1.0 | 0.9, 1.1 | 0.9* | 0.9, 1.0 | 1.0 | 0.9, 1.1 | 0.9*** | 0.9, 0.9 | 0.9** | 0.8, 0.9 | 0.8* | 0.6, 1.0 |
| **Number of ANC visits (ref = 4 or more)** | | | | | | | | | | | | |
| 0-3 visits[1] | 1.1 | 1.0, 1.2 | 1.0 | 0.9, 1.1 | 1.0 | 1.0, 1.1 | 1.0 | 1.0, 1.1 | 1.2* | 1.0, 1.3 | 1.0 | 0.9, 1.1 |
| **Size at birth (ref = normal)** | | | | | | | | | | | | |
| Small or very small | 1.0 | 0.9, 1.2 | 1.0 | 0.9, 1.1 | 1.1 | 0.9, 1.2 | 1.1** | 1.0, 1.2 | 1.2 | 1.0, 1.5 | 1.0 | 0.9, 1.2 |
| Large or very large | 1.0 | 0.9, 1.2 | 1.0 | 0.9, 1.2 | 1.1 | 0.9, 1.3 | 1.1* | 1.0, 1.2 | 1.1 | 0.8, 1.3 | 1.0 | 0.8, 1.2 |
| **Sex of child (ref = male)** | | | | | | | | | | | | |
| Female | 1.0 | 0.9, 1.1 | 1.1 | 1.0, 1.1 | 0.9 | 0.9, 1.0 | 1.0 | 1.0, 1.0 | 0.9 | 0.8, 1.0 | 1.0 | 0.9, 1.1 |
| **Parity (ref = 1)** | | | | | | | | | | | | |
| 2-3 | 0.7*** | 0.6, 0.8 | 0.9 | 0.9, 1.0 | 0.9** | 0.8, 1.0 | 0.9* | 0.9, 1.0 | 0.8*** | 0.7, 0.9 | 0.8* | 0.7, 1.0 |
| 4+ | 0.6*** | 0.5, 0.7 | 0.9* | 0.8, 1.0 | 1.0 | 0.9, 1.1 | 0.9*** | 0.8, 0.9 | 0.8*** | 0.7, 0.9 | 1.0 | 0.8, 1.2 |
| **Marital status** | | | | | | | | | | | | |
| Not married[1] | 0.9 | 0.7, 1.2 | 0.9* | 0.8, 1.0 | 1.1 | 0.9, 1.2 | 1.0 | 0.9, 1.0 | 0.9 | 0.8, 1.1 | 1.3* | 1.0, 1.6 |
| **Exposed to TV, radio, or newspaper (ref = < once per week** | | | | | | | | | | | | |
| At least once a week | 1.0 | 0.9, 1.1 | 1.1 | 1.0, 1.2 | 0.9 | 0.8, 1.0 | 1.0 | 1.0, 1.1 | 0.9 | 0.8, 1.1 | 1.0 | 0.8, 1.1 |
| **Employment (ref = not employed)** | | | | | | | | | | | | |
| Employed | 1.1 | 1.0, 1.2 | 1.0 | 0.9, 1.1 | 1.1 | 1.0, 1.2 | 1.0 | 1.0, 1.1 | 1.0 | 0.9, 1.1 | 0.8** | 0.7, 0.9 |
| **Education (ref = none or primary)** | | | | | | | | | | | | |
| Secondary or higher | 0.9 | 0.7, 1.0 | 1.0 | 0.9, 1.1 | 1.0 | 0.9, 1.1 | 1.0 | 1.0, 1.1 | 1.1* | 1.0, 1.2 | 1.2 | 1.0, 1.3 |
| **Wealth (ref = Lowest)** | | | | | | | | | | | | |
| Second | 1.0 | 0.9, 1.1 | 0.9 | 0.8, 1.0 | 1.0 | 0.9, 1.1 | 1.0 | 0.9, 1.0 | 1.1 | 0.9, 1.2 | 1.1 | 1.0, 1.4 |
| Middle | 1.0 | 0.9, 1.2 | 0.9 | 0.8, 1.0 | 1.0 | 0.9, 1.2 | 1.0 | 0.9, 1.1 | 1.0 | 0.9, 1.2 | 1.3* | 1.0, 1.6 |
| Fourth | 1.0 | 0.8, 1.2 | 1.0 | 0.8, 1.1 | 1.0 | 0.9, 1.2 | 0.9 | 0.9, 1.0 | 1.0 | 0.8, 1.3 | 1.4* | 1.0, 1.8 |
| Highest | 1.0 | 0.7, 1.3 | 0.9 | 0.8, 1.1 | 1.0 | 0.8, 1.1 | 0.9* | 0.8, 1.0 | 1.0 | 0.8, 1.4 | 1.4* | 1.0, 1.9 |
| **Place of residence (ref = urban)** | | | | | | | | | | | | |
| Rural | 1.0 | 0.8, 1.1 | 1.1 | 1.0, 1.2 | 1.0 | 0.9, 1.1 | 1.0 | 1.0, 1.1 | 0.9 | 0.8, 1.1 | 1.2 | 0.9, 1.4 |

Note: *p<0.05, **p<0.01, ***p<0.001; region not shown.

Supplement to: Mallick L, Wang W, Farid S, Pullum T. Initiation of Breastfeeding in Low- and Middle-Income Countries: A Time-to-Event Analysis. *Glob Health Sci Pract*. 2021;9(2). https://doi.org/10.9745/GHSP-D-20-00361

Supplement Figure 1a. Time to initiation of breastfeeding by place and mode of delivery, Europe and Asia

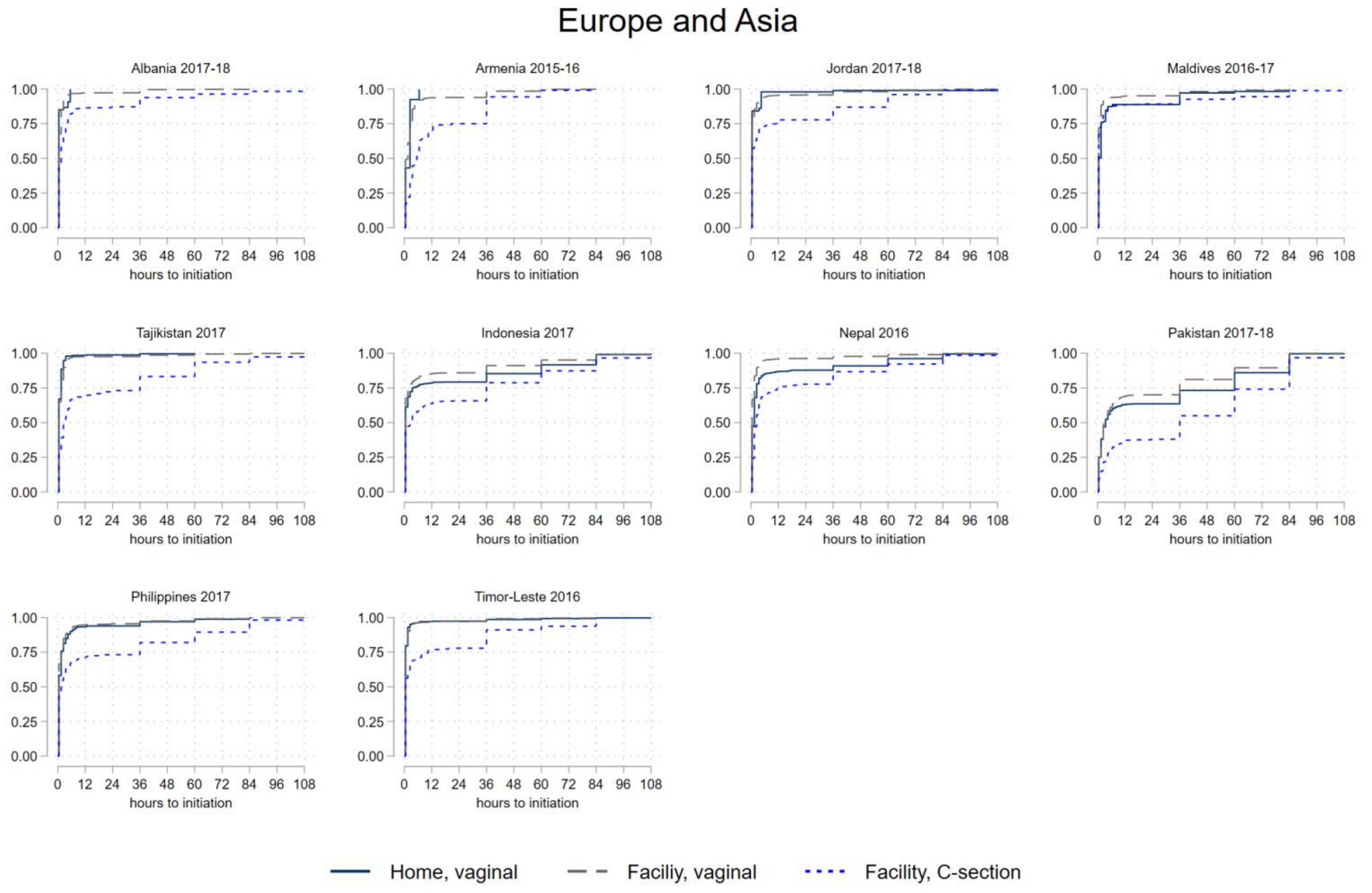

Supplement to: Mallick L, Wang W, Farid S, Pullum T. Initiation of Breastfeeding in Low- and Middle-Income Countries: A Time-to-Event Analysis. *Glob Health Sci Pract*. 2021;9(2).
**https://doi.org/10.9745/GHSP-D-20-00361**

Supplement Figure 1b. Time to initiation of breastfeeding by place and mode of delivery, sub-Saharan Africa and Haiti

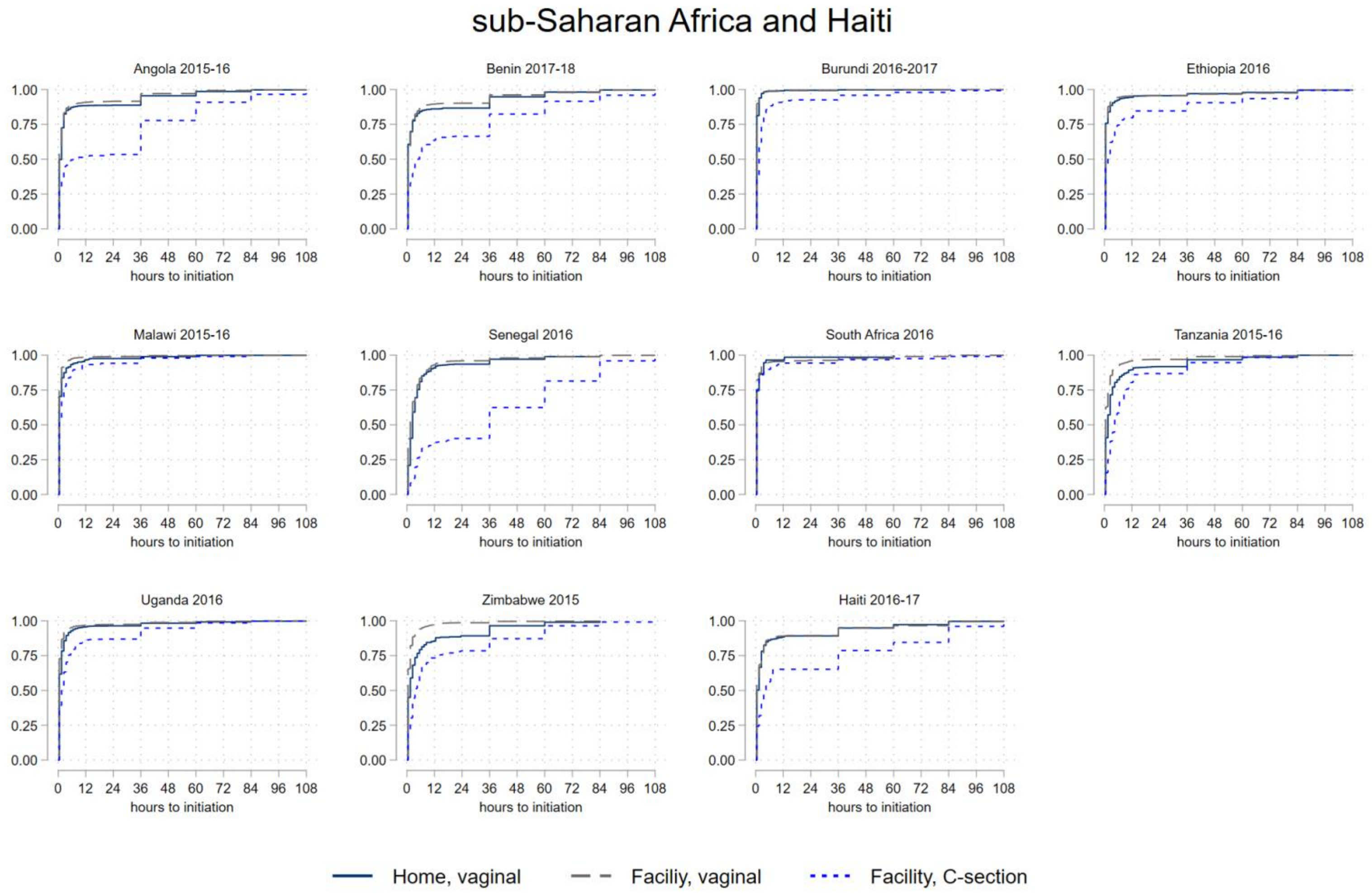

Supplement to: Mallick L, Wang W, Farid S, Pullum T. Initiation of Breastfeeding in Low- and Middle-Income Countries: A Time-to-Event Analysis. *Glob Health Sci Pract*. 2021;9(2). https://doi.org/10.9745/GHSP-D-20-00361

Supplement Figure 2a. Time to initiation of breastfeeding by immediate skin-to-skin contact, Europe and Asia

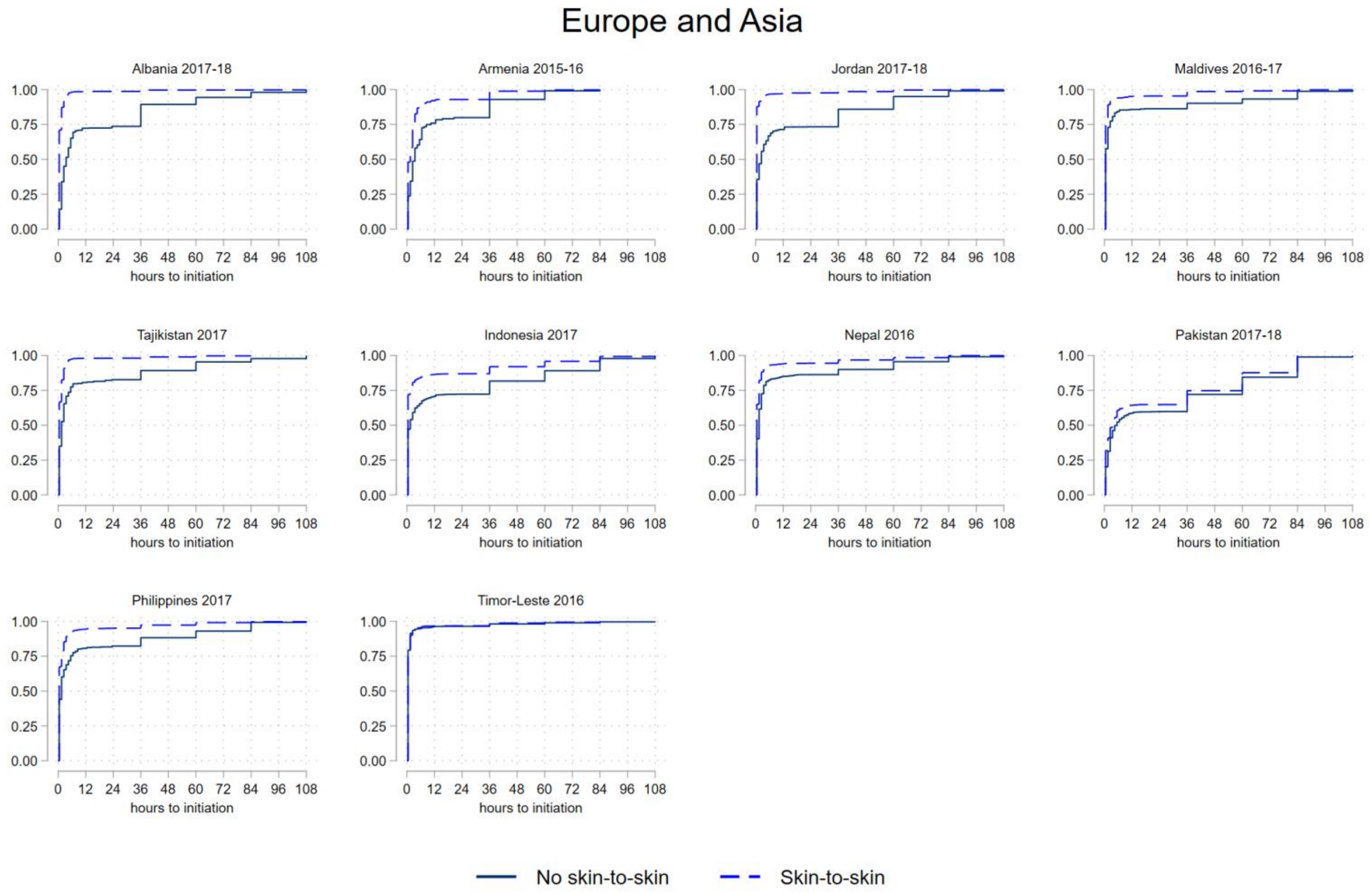

Supplement to: Mallick L, Wang W, Farid S, Pullum T. Initiation of Breastfeeding in Low- and Middle-Income Countries: A Time-to-Event Analysis. *Glob Health Sci Pract*. 2021;9(2).
**https://doi.org/10.9745/GHSP-D-20-00361**

Supplement Figure 2b. Time to initiation of breastfeeding by immediate skin-to-skin contact, sub-Saharan Africa and Haiti

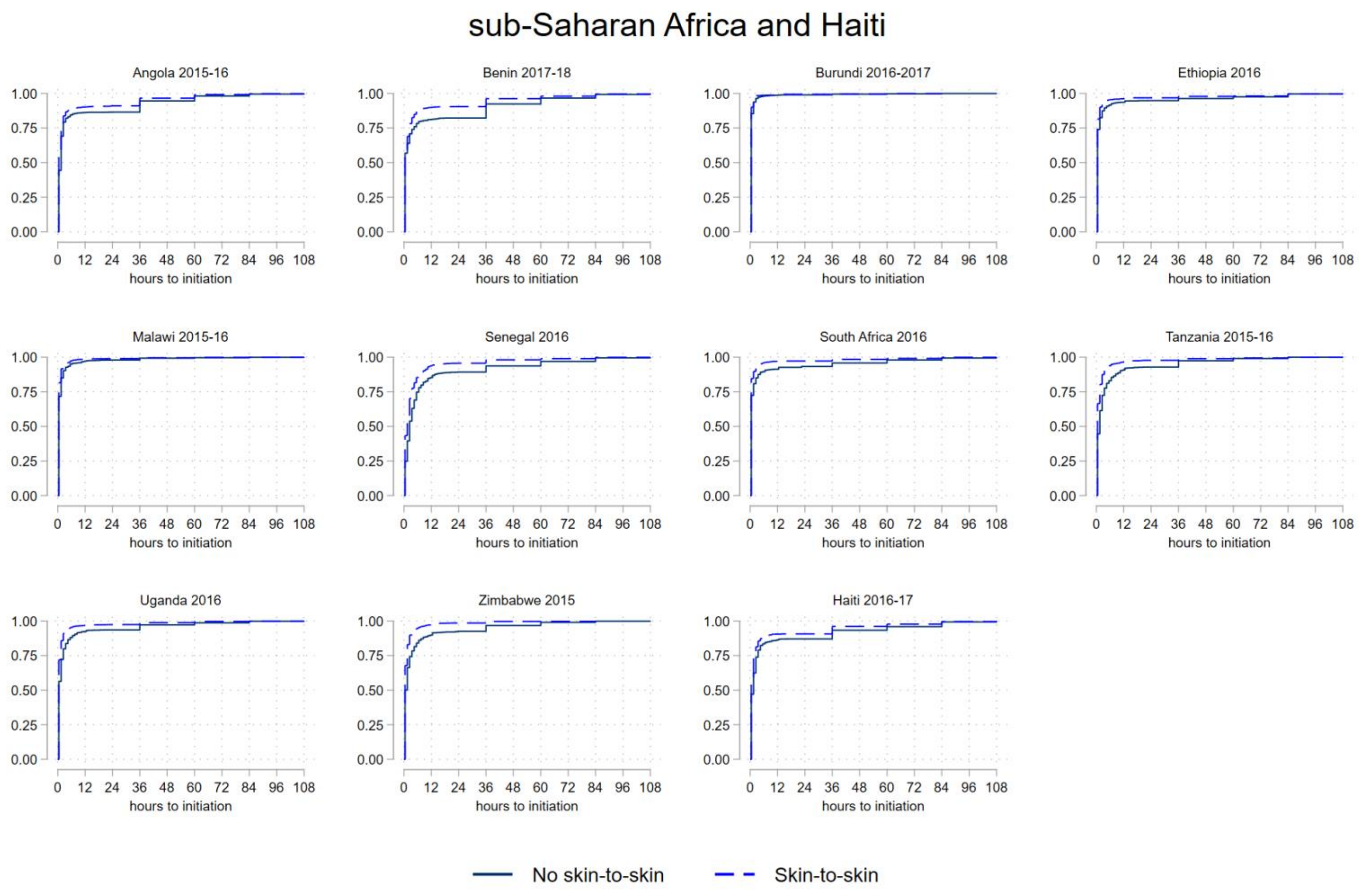

Supplement: 20-00361-Mallick-Supplement.pdf [file 20-00361-Mallick-Supplement.pdf]
